# Tau-proximity ligation assay reveals extensive previously undetected pathology prior to neurofibrillary tangles in preclinical Alzheimer’s disease

**DOI:** 10.1186/s40478-020-01117-y

**Published:** 2021-01-28

**Authors:** Nora Bengoa-Vergniory, Elisavet Velentza-Almpani, Ana Maria Silva, Connor Scott, Mariana Vargas-Caballero, Magdalena Sastre, Richard Wade-Martins, Javier Alegre-Abarrategui

**Affiliations:** 1 Department of Physiology, Anatomy and Genetics, University of Oxford, South Parks Road, Oxford, OX1 3QX UK; 2 Medical Research Council Centre for Molecular Bacteriology and Infection, Department of Infectious Disease, Imperial College London, Armstrong Road, London, SW7 2AZ UK; 3 Nuffield Department of Clinical Neurosciences, University of Oxford, Level 1, West Wing, John Radcliffe Hospital, Oxford, OX3 9DU UK; 4 Oxford Parkinson’s Disease Centre, University of Oxford, South Parks Road, Oxford, OX1 3QX UK; 5 School of Biological Sciences, University of Southampton, Southampton, SO17 1BJ UK; 6 Department of Brain Sciences, Imperial College London, Hammersmith Hospital, London, W12 0NN UK

**Keywords:** Tau, Alzheimer’s, Phosphorylation, AT8, Aggregation, Proximity-ligation assay, Early pathology, Multimer

## Abstract

**Background:**

Multimerization is a key process in prion-like disorders such as Alzheimer’s disease (AD), since it is a requirement for self-templating tau and beta-amyloid amyloidogenesis. AT8-immunohistochemistry for hyperphosphorylated tau is currently used for the diagnosis and staging of tau pathology. Given that tau–tau interactions can occur in the absence of hyperphosphorylation or other post-translational modifications (PTMs), the direct visualization of tau multimerization could uncover early pathological tau multimers.

**Methods:**

Here, we used bimolecular fluorescent complementation, rapamycin-dependent FKBP/FRB-tau interaction and transmission electron microscopy to prove the in vitro specificity of tau-proximity ligation assay (tau-PLA). We then analyzed *MAPT* KO and P301S transgenic mice, and human hippocampus and temporal isocortex of all Braak stages with tau-PLA and compared it with immunohistochemistry for the diagnostic antibody AT8, the early phosphorylation-dependent AT180, and the conformational-dependent antibody MC1. Finally, we performed proteinase-K treatment to infer the content of amyloidogenic beta-sheet fold.

**Results:**

Our novel tau-proximity ligation assay (tau-PLA) directly visualized tau–tau interactions in situ, and exclusively recognized tau multimers but not monomers. It elicited no signal in *MAPT* KO mouse brains, but extensively labelled P301S transgenic mice and AD brain. Two groups of structures were detected, a previously unreported widespread small-sized diffuse pathology and large, neurofibrillary-like lesions. Tau-PLA-labelled diffuse pathology appeared from the earliest Braak stages, mostly unaccompanied by tangle-like tau-immunohistochemistry, being significantly more sensitive than any small-sized dot-/thread-like pathology labelled by AT180-, AT8- and MC1-immunohistochemistry in most regions quantified at stages 0-II. Tau-PLA-labelled diffuse pathology was extremely sensitive to Proteinase-K, in contrast to large lesions.

**Conclusions:**

Tau-PLA is the first method to directly visualize tau multimers both in vitro and in situ with high specificity. We find that tau multimerization appears extensively from the earliest presymptomatic Braak stages as a previously unreported type of diffuse pathology. Importantly, in our study multimerization is the earliest detectable molecular event of AD tau pathology. Our findings open a new window to the study of early tau pathology, with potential implications in early diagnosis and the design of therapeutic strategies.

**Electronic supplementary material:**

The online version of this article (10.1186/s40478-020-01117-y) contains supplementary material, which is available to authorized users.

## Background

The early assessment of AD pathology is challenging: the disease is largely undetected until the first signs of cognitive impairment manifest, and validated biomarkers are too costly and complex to apply systematically [4]. Understanding the early cellular alterations is crucial if we are to design effective disease-modifying therapies to prevent neuronal loss. Our current understanding of the pathology of the early stages of disease derives from post-mortem studies of healthy individuals thought to have developed AD if they had lived longer. The two main neuropathologic features of AD, the accumulation of beta-amyloid in senile plaques (SPs) and microtubule associated protein tau (tau) in neurofibrillary tangles (NFTs) are variably labelled by silver stains [63], which allowed Braak and Braak to describe their progression through stereotyped neuroanatomical stages [7], with NFTs starting from the localized transentorhinal stage I. In contrast to SPs, the load of NFTs shows good correlation with clinical severity, duration of disease and neuronal loss [2, 3, 25]. NFTs contain fibrillar tau [13, 57, 67], which on transmission electron microscopy (TEM) appears as paired helical filaments (PHFs) and straight filaments (SFs) [38]. At least a proportion of fibrillar tau is abnormally phosphorylated at multiple residues [26, 30] and antibodies against phosphorylated tau epitopes such as AT8 have replaced silver staining for diagnosis and staging [6]. However, neuronal loss is superior to the number of NFTs, suggesting undetected tau species or other insults mediate toxicity [25]. Accumulating evidence indicates that early tau aggregates/multimers are the true toxic species that are responsible for the prion-like spread of tau pathology [28, 36, 54]. This also suggests a proportion of tau pathology is not being recognised by immunohistochemistry against hyperphosphorylated tau (e.g. AT8), particularly since fibrillization and seed propagation of tau can happen independently of phosphorylation [20, 22, 23, 27, 34, 48, 52, 68]. Recently developed biochemical and cellular assays prove that tau multimerization occurs early in the development of tau pathology and correlates with specific tau PTMs events like phosphorylation in Thr321 or early conformational changes of tau detected by AT180 and MC1 antibodies respectively [18, 37].

Given that tau multimerization is a pivotal step in the development of abnormal tau aggregates, we hypothesized that the ability to directly visualize this molecular event in situ, or in other words, to visualize tau–tau interactions, could provide a window into the early pathogenic stages of AD. In this study, we have developed a new technique, tau-PLA, which only recognizes tau–tau interactions without recognizing monomers in vitro. In addition to staining neurofibrillary-like lesions in human brain, tau-PLA detects an early and previously unreported type of small-sized diffuse pathology. We have found that this diffuse pathology builds up extensively from the earliest AD pathological phases in previously unrecognized medial temporal/hippocampal areas well before the appearance of NFT and neuropil thread (NT) pathology detected by conventional tau immunohistochemistry. We believe our findings shed new light and open potential new avenues into the investigation of early pathological processes of AD.

## Materials and methods

### Cell culture

HEK293 cells were cultured in Dulbecco’s modified Eagle’s medium (DMEM) supplemented with 10% fetal bovine serum (FBS), 2 mM glutamine and 100 U/ml penicillin/streptomycin (Thermo Fisher Scientific). Cells were maintained at 37 °C in a 5% CO_2_ humidified atmosphere.

### Vector construction

The tau bimolecular fluorescence complementation (BiFC) constructs (pmGFP10C-tau and pmGFP11C-tau) were a kind gift from Henri Huttunen (Addgene plasmids #71433 and # 71434 [9]). To generate FK506 binding protein (FKBP)-tau and FK506 rapamycin binding (FRB)-tau constructs, tau (0N4R) was amplified from pmGFP10C-tau using primers 5′-GGCGGCTCGAGCGCCACCATGGATGTATTCATGGCTGAGCCCCGCCAGGAGTTCGAAGTG-3′ and 5′- ATCCTCTTCTGAGATGAGTTTTTGTTCGAATCTAGATCACAAACCCTGCTTGGCCAGGGAGGCAGA-3′ and NEBuilder HiFi DNA Assembly Master Mix (New England BioLabs), according to the manufacturer’s instructions. Alpha-synuclein was excised from the previously described [53] FKBP-alpha-synuclein and FRB-alpha-synuclein plasmids and replaced by tau. Successful construction was confirmed by colony-PCR and DNA sequencing.

### Tau BiFC assay

HEK293 cells grew on poly-l-lysine-treated coverslips in 24-well plates overnight at a density of 2 × 10^5^ cells, transiently transfected with 100 ng of pmGFP10C-tau and pmGFP11C-tau using Lipofectamine-2000 and Plus reagents (Thermo Fisher Scientific), imaged to detect GFP expression and fixed in 4% paraformaldehyde (PFA) for tau-PLA experiments or harvested for western blot (WB) 24 h after transfection.

### Tau FKBP-FRB-rapamycin assay

The tau FKBP-FRB-rapamycin assay was performed as described previously [53]. HEK293 cells were transfected with 25 ng of FKBP-tau and FRB-tau, incubated at 37 °C for 4 h, washed with phosphate-buffered saline (PBS), treated with or without 400 nM rapamycin (Calbiochem) for 1 h, washed with PBS and fixed in 4% PFA for tau-PLA experiments. Images of fixed cells were obtained using the DV Elite system based on an Olympus IX71 fully motorized widefield deconvolution inverted microscope with a 60× objective 1.40 numerical aperture (NA) fitted with a CoolSNAP HQ2 cooled charge-coupled device (CCD) camera (Photometrics) driven by SoftWoRx 5.0 software (Applied Precision). Several cell positions were chosen randomly and recorded using the motorized stage. z-stacks were acquired at 0.250 µm intervals to cover the entire volume of each cell. Quantification of tau-PLA puncta was performed on deconvolved images using Fiji [55]: tau-PLA signals were enhanced using a median filter and then separated from the background using Otsu’s method of thresholding. Signals were detected and counted.

### Analysis of tau protein

For WB analysis of tau-BiFC protein, HEK293 cells transiently transfected with pmGFP10C-tau and pmGFP11C-tau were washed in PBS and lysed in RIPA buffer (50 mM Tris pH 8, 150 mM NaCl, 2 mM EGTA, 0.5% sodium deoxycholate, 1% Igepal, 0.1% sodium dodecyl sulphate (SDS)] with protease inhibitors (Complete Mini, EDTA-free, Roche). Cells were disrupted by repeated pipetting followed by sonication on ice. Cell lysates were pelleted at 2000 rpm for 10 min at 4 °C. Protein concentration in the supernatant was determined by BCA assay, according to the manufacturer’s instructions (Sigma). Ten micrograms of protein from cell lysates were reduced in Laemmli buffer and heated to 95 °C for 10 min for SDS–polyacrylamide gel electrophoresis (PAGE) or resuspended in SDS-free Novex™ Tris–Glycine Native Sample Buffer (Thermo Fisher Scientific) for non-denaturing PAGE. Proteins were separated on pre-cast Bio-Rad Criterion™ TGX™ 4–15% gradient gels. After transfer of proteins to polyvinylidene difluoride (PVDF) membranes (Bio-Rad) and blocking in 5% (w/v) powdered skimmed milk in Tris-buffered saline/0.1% Tween 20 (TBS-T), membranes were incubated overnight at 4 °C in primary antibody (tau5, Abcam) diluted in 1% skimmed milk/TBS-T. After washing three times in TBS-T, the membrane was incubated with HRP-conjugated IgG goat anti-mouse secondary antibody (Bio-Rad) for 1 h at room temperature (RT). The membrane was washed again in TBS-T and the signal visualized with ECL reagent (Millipore) and exposure in the ChemiDoc™ Touch Imaging System (Bio-Rad).

### Recombinant tau and alpha-synuclein assembly

Two mg/ml of alpha-synuclein (rPeptide) was shaken at 37 °C and 250 rpm for 120 h to induce fibrillar aggregation. Forty µM tau-441 2N4R (rPeptide) was shaken in sodium acetate (Sigma) 100 mM pH 7.0, 40 µM Heparin (Sigma), 2 mM DTT (Thermo) at 37 °C and 250 rpm for either 16 or 360 h to induce aggregation, as per [50] (modified). Tau monomer was filtered through an Amicon 100 kDa molecular weight cut-off filter column (Sigma) prior to use to remove any aggregated species. Samples were subsequently frozen until further analysis.

### TEM

Ten µl of 40 µM samples for tau and 1 mg/ml for alpha-synuclein were applied to carbon coated TEM grids (TAAB) after glow discharge, incubated for 2 min on the grids at RT, stained by uranyl acetate 2% for 10 s, dried immediately and stored at RT. Images were acquired with a FEI Tecnai 12 TEM microscope (120 kV) with a Gatan US1000 camera.

### Animal tissue

*MAPT* KO mice missing exon 1 in the *MAPT* gene after replacement with the neomycin resistant cassette [14, 64], transgenic P301S tau mice expressing the shortest human four-repeat tau isoform (0N4R) with the P301S mutation under the control of the murine *thy1* promoter [1], and wild-type aged matched littermates C57BL/6, were used in this study. Six month old *MAPT* KO (N = 6), P301S (N = 6), and C57BL/6 (N = 6) mice, together with 3 (N = 2) and 9 (N = 2) month old P301S mice were deeply anesthetized and perfused transcardially with phosphate buffered saline (PBS). Brains were extracted and fixed in 4% paraformaldehyde in PBS for 24 h at 4 °C, followed by paraffin embedding and microtome sectioning.

### Human tissue

Formalin-Fixed Paraffin-Embedded (FFPE) 5 µm thick slices of posterior hippocampus (lateral geniculate nucleus level) from individuals without neurological disease or *intra vitam* diagnosis of AD were supplied by the Oxford Brain Bank and Multiple Sclerosis and Parkinson’s Tissue Bank of Imperial College London (Additional file 1: Tables S1 and S2).

### In situ proximity ligation assay (PLA)

Tau-PLA experiments were performed as described previously with minor modifications [53], using the Duolink kits (Sigma) according to the manufacturer’s instructions. The conjugates were prepared using the Duolink Probemaker Plus and Minus kits and tau5 antibody (ab80579, Abcam). For fluorescent tau-PLA, transfected HEK293 cells were fixed in 4% PFA. For brightfield tau-PLA, FFPE tissue was dewaxed in xylene, rehydrated via graded alcohols, treated with 10% H_2_O_2_ for 1 h at RT to block endogenous peroxidases, as previously described [53] and incubated with citrate buffer (pH 6) for heat-induced antigen retrieval. All samples were incubated with Duolink block solution at 37 °C for 1 h and then with tau conjugates diluted in Duolink PLA diluent (1:2000 for fluorescent tau-PLA and 1:250 for brightfield tau-PLA experiments) for 1 h at 37 °C, then overnight at 4 °C. Samples were washed with TBS-0.05% Tween 20 and incubated with Duolink ligation reagents for 1 h at 37 °C and then with Duolink amplification reagents for 2.5 h at 37 °C. For fluorescent tau-PLA experiments, samples were washed and mounted in Vectashield with 1 µg/ml DAPI counterstain. Tissue sections were washed and incubated with a Duolink detection solution for 1 h at RT followed by a Duolink substrate solution for 20 min at RT. Tissue sections were then counterstained with hematoxylin, dehydrated in graded alcohols and xylene and mounted with DPX mounting reagent.

For tau-PLA co-immunofluorescence, immunofluorescence was performed after antigen retrieval and before PLA block. This process consisted of 1 h RT incubation in primary antibody (GFAP Z0334, Sigma; Iba1 019-19741, Wako; Tau5, Abcam; amyloid-beta ab11132, Abcam), after which slides were washed with TBS-0.05% Tween 20. Slides were then incubated for 1 h RT with secondary antibodies (Alexa488 or Alexa680, Life Technologies) and then washed with TBS-0.05% Tween 20.

For recombinant protein analysis, 10 µl of 40 µM samples (1 mg/ml for alpha-synuclein fibrils) were spotted on poly-l-lysine coated coverslips, left at RT for 30 min, then PFA-fixed for 10 min and treated as above for fluorescent tau-PLA.

### Immunohistochemistry and proteinase K (PK) treatment

FFPE tissue was dewaxed in xylene, rehydrated in graded alcohols and then blocked with 10% H_2_O_2_ for 1 h at RT in the dark. Antigen retrieval was performed via microwave heating with citrate buffer pH-6 (tau5, AT180, anti-6X His tag), immersion in 80% formic acid for 1 h (4G8) or no treatment (AT8, MC1). Then sections were blocked with 10% normal goat serum in TBS-0.1% Triton X-100 for 1 h at RT, incubated with tau5 (1:200), anti-beta amyloid clone 4G8 (Biolegend, 1:2000), AT8 (Innogenetics, 1:500), AT180 (ThermoFisher Scientific, 1:1000), MC1 (kindly provided by Peter Davies, The Feinstein Institute for Medical Research, Manhasset, NY, 1:100), or anti-6X His tag (Abcam, 1:200) at 4 °C overnight, incubated with biotinylated goat anti-mouse/anti-rabbit IgG secondary antibody (Jackson Immunoresearch) for 1 h at RT, washed in TBS-0.1% Triton X-100, incubated with VectaStain ABC reagent (Vector Labs) for 1 h at RT, treated with 3,3′-Diaminobenzidine (DAB, Sigma) for 3 min, counterstained with hematoxylin, dehydrated in graded alcohols and xylene and mounted with DPX mounting reagent.

PK treatment was performed as an additional step after antigen retrieval by incubating slides in 50 µg/ml PK (Sigma) 10 mM Tris HCl pH 7.8, 100 mM NaCl, 0.1% NP-40 at 37 °C for the stated times (0 s, 10 s, 1 min and 2 min).

### Imaging and neuropathological analysis

Slides were imaged blinded to the researchers with an Aperio-Scanscope (40x objective). Three representative images were blindly and manually taken per analysed region from the scan and run through ImageJ blindly until obtaining the final scoring for each slide. Images were color-thresholded and analysed for particle detection (Additional file 1: Fig. S1A and B). Particle size was adjusted for the object of analysis: large perikaryal lesions of coalescent signal, mostly representing NFTs, were adjusted to 12.5–100, SPs were adjusted to 12.5–10,000 and diffuse tau-PLA signal labelling of small structures was adjusted to 1.5–3.5 µm^2^. The mean per sample and region was calculated and subsequently analysed. Semi-quantitative analysis was performed by blindly taking and rating 3 different 40 x images for every region according to a semi-quantitative scale (Additional file 1: Fig. S1C) between 1 and 6. Unpaired two-tailed Student’s *t* test and one-way ANOVA with Dunnett’s post hoc test were performed using GraphPad Software for the statistical analysis and comparison of the groups. Statistical significance was defined as p < 0.05 for all analyses.

## Results

### Tau-PLA detects tau multimerization in vitro

**Fig. 1:**
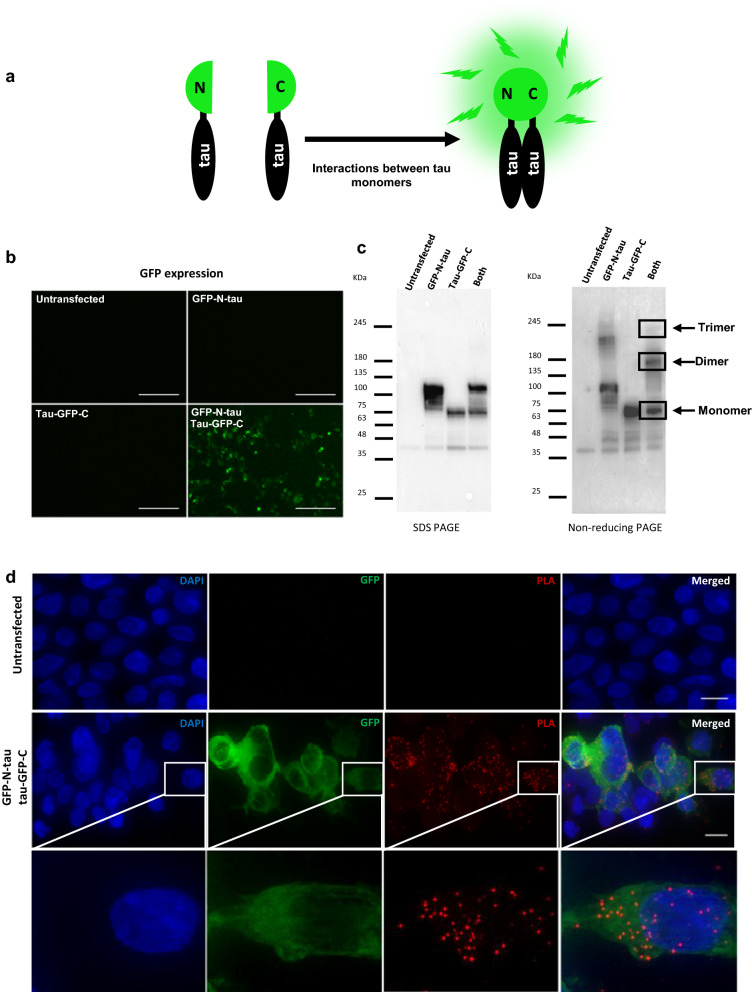
Tau-PLA detects tau-BiFC complexes. **a** Tau-BiFC constructs contain non-fluorescent halves of GFP fused to tau. GFP activity is detected only when tau molecules interact between them and the split GFPs fold together. **b**, **c** Expression of tau-BiFC constructs in HEK293 cells was analysed by fluorescent microscopy and western blot. Tau-BiFC constructs form oligomeric species, as shown by non-denaturing PAGE. Scale bar: 100 µm. **d** Green fluorescence in HEK293 cells expressing tau-BiFC constructs indicated tau–tau interactions. Tau-PLA signal (red) was co-localized with the BiFC signal (green) in transfected cells, indicating that tau-PLA detects tau–tau interactions. Nuclei were identified by DAPI staining (blue). Images are maximum projections of z-stacks. Scale bar: 10 µm. All experiments were performed in triplicate

Protein multimerization is a fundamental molecular requisite for proteins such as tau, amyloid-beta or alpha-synuclein to seed pathology. In order to detect tau–tau interactions in situ, we took advantage of the knowledge we generated developing the alpha-synuclein proximity ligation assay for Parkinson’s disease [53], namely, the ability of PLA to detect protein interactions of two or more molecules, but not monomers, when conjugating the same epitope blocking monoclonal antibody to PLUS and MINUS PLA probes. The assay can therefore only result in productive ligation when at least two tau molecules are in close proximity. Ligation allows rolling circle amplification to produce a globular DNA thread which is then visualized with labelled oligonucleotides as a discrete dot. Therefore, PLA signal is dotted, and each dot represents a recognized interaction event. Since we aimed to detect tau multimerization regardless of its PTMs, to allow visualization of potential tau–tau interactions ahead of phosphorylation and conformational changes, we chose tau5, a pan-tau monoclonal antibody against the human tau sequence 218–225 [51], not phosphorylated in vivo, to build the tau-PLA assay. To validate our new PLA technique, we used several in vitro methods. First, we used a previously validated [9] bimolecular BiFC assay which requires the co-transfection of two plasmids encoding tau fused to a split GFP reporter (Fig. 1a). In the absence of one of the plasmids, only one half of GFP is expressed and therefore fluorescence is impaired, however when the two copies are overexpressed by co-transfection, tau multimerization brings the GFP halves into proximity, allowing them to fold and producing fluorescence in HEK293 cells, as is shown in Fig. 1b. The formation of tau high molecular species was confirmed by non-reducing gel electrophoresis (Fig. 1c). Tau-PLA signal was prevalent in cells exhibiting BiFC fluorescence (Fig. 1d).

**Fig. 2:**
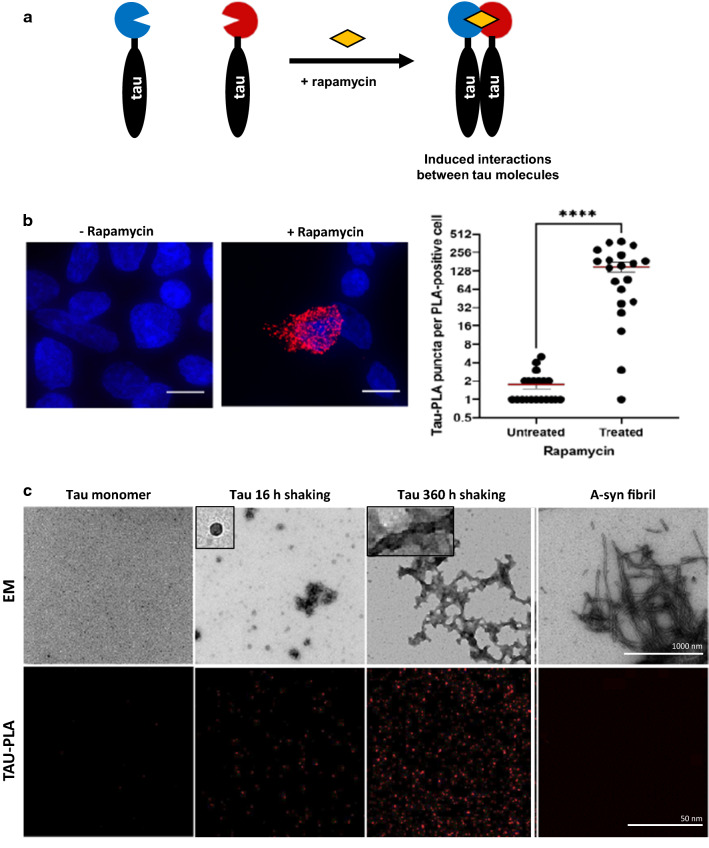
Tau-tau interaction is detected by tau-PLA. **a** Inducible homomeric tau complexes are detected by tau-PLA. Tau was fused to FKBP or FRB which form a ternary complex with rapamycin. Tau complexes are forced to conditionally associate in the presence of rapamycin. **b** HEK293 cells were transfected with both constructs and incubated with or without rapamycin for 1 h after transfection (left). Quantification of tau-PLA puncta (right). Tau-PLA puncta significantly increased in the presence of rapamycin, as determined by unpaired two-tailed Student’s t-test (*p < 0.05), indicating tau-PLA detects tau–tau interactions. Data are mean ± SEM, N = 20 cells per condition. Nuclei were identified by DAPI staining (blue). Images are maximum projections of z-stacks. Scale bar: 10 μm. All experiments were performed in triplicates. **c** Tau-PLA detects tau–tau interaction of recombinant tau but not monomeric tau. Aggregation of recombinant full-length unphosphorylated tau4R was induced by shaking in the presence of heparin. After 16 h of shaking, both amorphous and spherical/globular tau accumulations are seen on TEM analysis. After 360 h of shaking tau assembled into a mixed population of shaped species, including globular structures and filaments, mostly of short or intermediate length. Alpha- synuclein assembled into fibrils after 120 h of shaking (without heparin). The obtained recombinant protein preparations were subjected to TEM (all images at 18.500 x) and tau-PLA

Next, we fused tau to either FKBP or FRB (Fig. 2a), an inducible system where tau–tau interactions are dependent on the addition of rapamycin [44]. In contrast with the BiFC assay, in which overexpression was used to ensure tau multimerization, we now optimized the experimental conditions to achieve low levels of expression (Additional file 1: Fig. S2). In these conditions, tau-PLA showed a negligible signal, despite the presence of tau monomers. Induction of tau–tau interactions by addition of rapamycin caused an increase in the numbers of tau-PLA puncta per cell (Fig. 2b). This suggests that tau-PLA detects intracellular tau–tau interactions, but not monomers.

Both the BiFC and FKBP/FRB systems are based on tagged tau constructs. To prove that recognition of tau–tau interactions and not tau monomers by tau-PLA did not depend on the tags, we used untagged full-length unphosphorylated recombinant 2N4R tau monomers and aggregates. Tau-tau interactions were obtained by the addition of heparin and shaking [23, 52] and the progress of tau complex formation was monitored with TEM (Fig. 2c). The tau monomeric preparation lacked any structure on TEM. After 16 h of shaking, spherical/globular tau accumulations started to form, and after 360 h of shaking tau assembled predominantly into filaments. A proportion of the globular structures were disposed in bundles or short chains. We also generated alpha-synuclein fibrils by shaking recombinant protein to demonstrate that tau-PLA is specific to tau and not to general aggregation. Imaging equal amounts of the preparations revealed that tau-PLA signal was highly specific for tau–tau interactions, while it did not recognize monomeric tau and alpha-synuclein fibrils (Fig. 2c). Together with our previous findings these results indicate that tau-PLA detects tau-multimers in vitro.

### In situ specificity of tau-PLA in transgenic and *MAPT* KO mice

To demonstrate the in situ specificity of the assay and to exclude the possibility of non-specific binding (background), analysis of animal brain tissue was performed. FFPE brain sections from 2 months-old *MAPT* KO mice [14, 64] were analysed with tau-PLA, alongside sections from 6 months-old P301S transgenic mice [1] and C57BL/6 age-matched controls. Tau-PLA analysis revealed strong staining in the P301S mice, with a significant level of PLA load on axonal tracts, while no signal was detected in *MAPT* KO or C57BL/6 control mice (Additional file 1: Fig. S3). A comparison with AT8 staining indicated that tau-PLA reveals prominent tau multimerization in anatomical brain areas devoid of AT8 staining such as CA1 region of hippocampus and striatum (Additional file 1: Fig. S3). Tau-PLA analysis also revealed that tau-PLA signal accumulates with age in P301S animals (Additional file 1: Fig. S3).

We also performed no-ligase controls to establish that the observed signal was a result of productive tau-PLA. Ligase removal from the tau-PLA assay resulted in absolute depletion of tau-PLA signal, indicating that the signal was indeed representative of tau–tau interacting molecules (Additional file 1: Fig. S4).

To further prove that tau-PLA is antibody-dependent, and it does not give false positive results due to PLA itself, we generated PLA probes conjugated with a 6xHisTag antibody. As we expected no signal was detected after performing immunohistochemistry with 6xHisTag antibody and 6xHisTag-PLA (Additional file 1: Fig. S4C). Altogether, these data support that tau-PLA can detect tau–tau interactions with high specificity and negligible background in situ.

### Tau-PLA labels a range of structures in AD brain, from large lesions to diffuse pathology

**Fig. 3:**
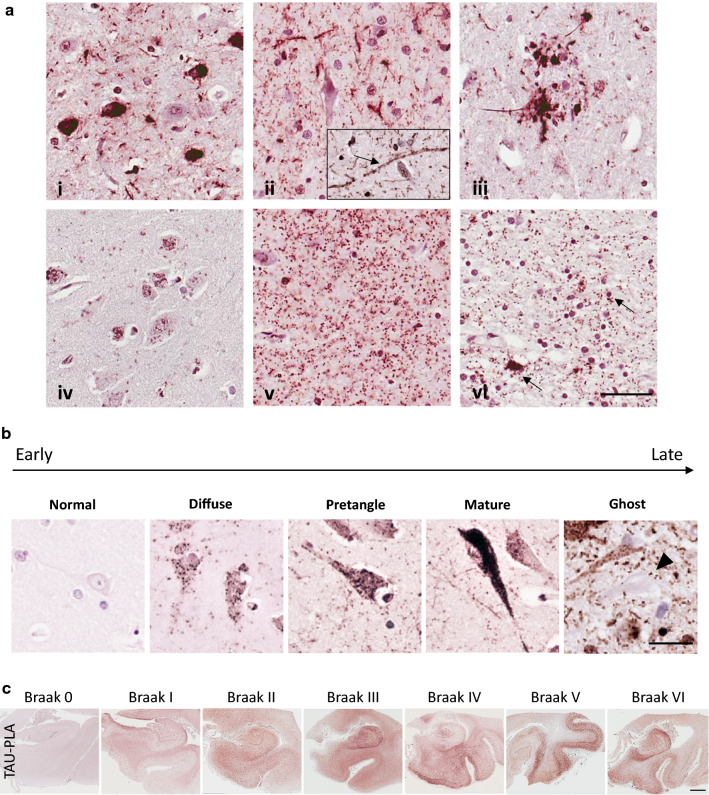
Tau-PLA highlights different staining patterns. Images taken from a stage V patient. **a** Tau-PLA labels (i) neurofibrillary tangles (CA1). Note also unlabelled neurons. (ii) Tau-PLA labels neuropil threads (entorhinal region). Arrow in inset shows alignment of tau-PLA dots onto elements with axonal morphology. (iii) Tau-PLA labels neurites within neuritic plaque (CA1). (iv) Tau-PLA labels morphologically intact neurons with accumulation of tau complexes (CA1). Note these appear in areas lacking any AT8 immunohistochemistry labelling and are different to pre-tangle neurons. (v) Tau-PLA labels diffuse accumulation of tau–tau complexes in the neuropil (CA4). (vi) Tau-PLA labels the perikarya of occasional oligodendrocytes (arrows, white matter). Scale bar 50 μm. **b** Neurofibrillary tangle maturity characterized by tau-PLA, from left to right examples of tau-PLA staining for normal, diffuse, pretangle, mature and ghost pathology. Scale bar 50 μm. **c** Tau-PLA distribution through the different Braak stages. Scale bar 2 mm

We next evaluated post-mortem human tissue using tau-PLA, in order to examine if tau-PLA recognizes endogenous disease-related tau–tau interactions. FFPE hippocampal post-mortem sections from 67 individuals of neurofibrillary Braak stages 0, I, II, III, IV, V and VI were analysed with tau-PLA and immunohistochemistry (Additional file 1: Table S1, S2). In AD brain (Braak stage V), tau-PLA revealed several staining patterns, indicating labelling of a breath of structures across hippocampal regions and temporal isocortex (Fig. 3). Together with neurofibrillary-like structures (NFTs, NTs and dystrophic neurites within neuritic plaques) that presented the same regional distribution as AT8/tau5-immunohistochemistry (Figs. 3ai–iii, 4, 5), tau-PLA also detected a previously unreported small-sized diffuse pathology comprised of tau multimers (Fig. 3aiv). This type of tau-PLA labelling consisted of individual non-coalescent puncta spreading out in the neuropil of the cortex, white matter and the soma of a proportion of morphologically intact neurons without NFTs (Fig. 3aiv–vi), either in the cytoplasm and sometimes around or inside the nucleus. Pretangle staining was also detected in the cytoplasm of intact neurons (Fig. 3aiv), which in borderline cases was difficult to differentiate from NFT labelling. When in neurons, the labelling of the diffuse pathology was different than pretangles; it was lighter, non-coalescent, and in areas where no pretangles were seen with AT8-immunohistochemistry. The neuronal labelling of tau-PLA revealed that tau multimerization starts early before neurofibrillary tangle maturity, with tau-PLA labelling being abolished at the level of lesions likely representing ghost tangles (Fig. 3b). In the subcortical white matter and occasionally in the cortex, the PLA dots were frequently aligned, sometimes into rail track-type structures, suggesting intra-axonal localization (Fig. 3aii arrow). The diffuse PLA tau pathology, which appeared largely unaccompanied by AT8 immunohistochemistry, is reminiscent of the diffuse neuropil pattern of alpha-synuclein oligomers in Parkinson’s disease [53]. Finally, tau-PLA labelled tau–tau interactions on the perikarya of scattered oligodendrocytes in the white matter (Fig. 3avi), a feature which we did not observe on AT8-immunohistochemistry.

**Fig. 4:**
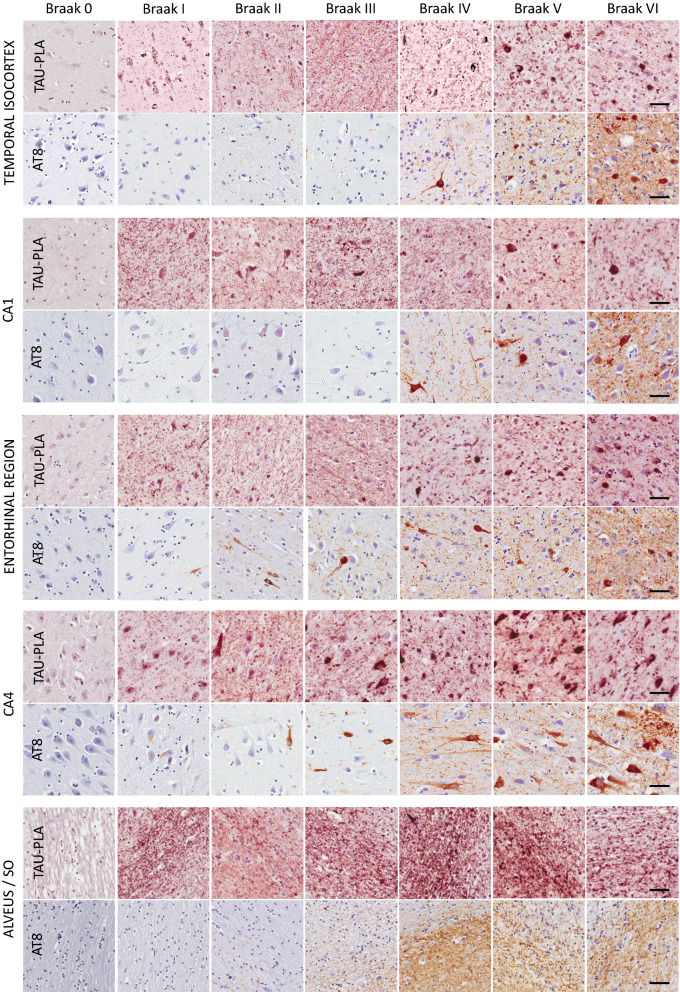
Early tau multimerization detection, prior to detection of tau hyperphosphorylation and misfolding across hippocampal regions and temporal isocortex. Representative images of selected hippocampal regions are shown here. FFPE sections of posterior hippocampus at the level of lateral geniculate nucleus from Braak 0 to VI were stained with tau-PLA and AT8-immunohistochemistry. Minimal PLA and immunohistochemistry signal is seen in Braak 0. Tau-PLA reveals abundant pathology in Braak stages I to III and onwards, while AT8-immunohistochemistry is absent or relatively low at initial stages, with the signal mostly appearing after Braak III. Scale bar 50 μm

**Fig. 5:**
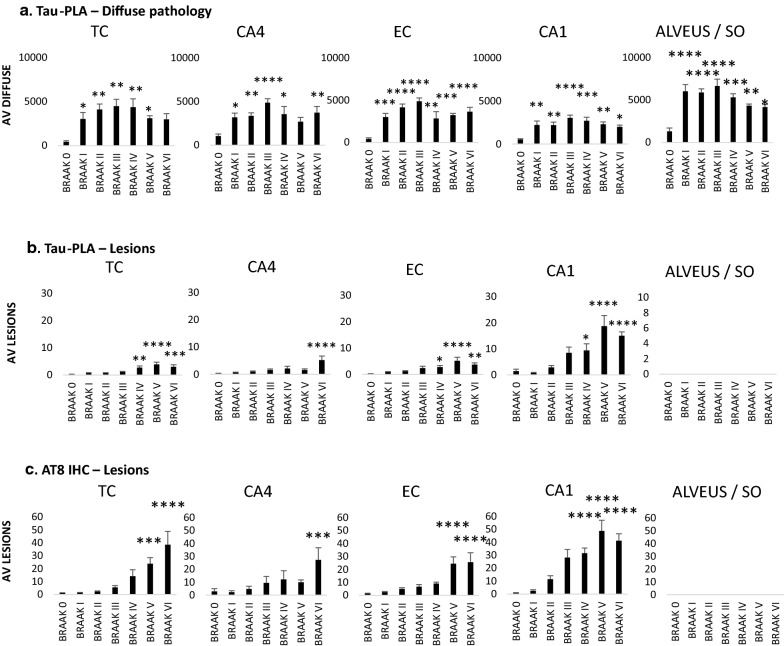
Quantification of tau-PLA labelled diffuse pathology and tau-PLA and AT8 IHC labelled large perikaryal neurofibrillary-type lesions in hippocampal regions. **a**–**c** Automated quantification of **a** tau-PLA diffuse signal, **b** tau-PLA lesions and **c** AT8-IHC lesions in different brain regions. All groups were compared to control (Braak 0) through a ONE-WAY ANOVA (Dunnet). N = 11/12/12/9/7/8/8. *p < 0.05, **p < 0.01, ***p < 0.001, ****p < 0.0001. *TC* temporal isocortex, *EC* entorhinal cortex, *SO* stratum oriens, *AV* average

Although a proportion of this diffuse pathology was clearly intraneuronal, by performing co-immunofluorescence of tau-PLA with GFAP and Iba1 (astrocytes and microglia, respectively Additional file 1: Fig. S5, we identified that a minority of the dots co-localized with these markers, suggesting that, in addition to neurons, a small proportion of these diffuse small complexes were in astrocytes and microglia.

### Extensive tau multimerization revealed by tau-PLA occurs widely and early in the development of AD tau pathology

We next focused on determining the spatiotemporal evolution across Braak staging comparing the different tau-PLA labelled structures with *bona fide* early tau-immunohistochemistry, namely that assessed through staining with AT8- and AT180 (for phosphorylated tau epitopes), and MC1 (misfolded tau).

Firstly, we compared brain sections from hippocampal regions and temporal isocortex stained with tau-PLA and AT8-immunohistochemistry (the “Gold Standard” for tau-immunohistochemistry in neuropathology) across all six Braak stages. As presented above, the main staining patterns of tau-PLA can be divided into two; large lesions of perikaryal NFT-like structures filled with coalescent dots, and small-sized diffuse pathology, which was labelled by discrete non-clustered dots. We used computer-aided quantification of both types of pathology.

In Braak stage 0 cases, a negligible number of tau-PLA labelled perikaryal lesions and minimal average diffuse pathology were identified (Figs. 3c, 4, 5). Tau5, the antibody used for tau-PLA, recognizes a phosphorylation independent epitope which enables the labelling of widespread physiological monomeric tau within the axon-neuropil domain of neurons when used with immunohistochemistry methods [62]. Our in vitro results indicated that tau5 is epitope blocking therefore preventing tau-PLA to recognize monomers and allowing the detection of tau multimers only. The minimal labelling by tau-PLA in Braak 0 cases, provides further evidence that tau-PLA does not detect endogenous tau monomers in human brain. However, it is important here to highlight that although the majority of Braak 0 cases appeared to have a negligible level of tau-PLA signal, a number of individuals presented a moderate to high load of tau-PLA in the hippocampal regions (Additional file 1: Fig. S6).

From Braak stage I onwards, tau-PLA recognized lesions paralleling the same distribution pattern as AT8-immunohistochemistry (Figs. 3c, 4, 5b, c), although lower in number, from small numbers limited to the transentorhinal cortex in Braak stage I to a progressively more widespread distribution up to Braak stage VI. It is worth noting that tau-PLA detected an almost identical number of lesions as tau5-immunohistochemistry (Fig. 5b, Additional file 1: S7) indicating the differences between AT8-immunohistochemistry and tau-PLA were due to the superiority of AT8 to tau5 antibody for lesions, in agreement with previous reports supporting that the binding of tau5 can be affected by the nearby phosphorylation sites [45].

Interestingly, tau-PLA detected extensive diffuse tau pathology as soon as Braak stage I, mostly unaccompanied by AT8-immunohistochemistry, in all studied regions (Fig. 3c, 4, 5a), in contrast to the scarce and anatomically restricted labelling of NFT-like pathology at early stages. This abrupt increase in all areas quantified from Braak I was reduced but still significant at later stages (Braak IV, V and VI) (Figs. 3c, 4, 5a) and was especially pronounced in the alveus/stratum oriens. In agreement with our data, AT8-immunoreactivity was previously reported to appear in the alveus in National Institute on Aging/Alzheimer Association (NIA/AA) stages B2 and B3 of neurofibrillary degeneration [49] (corresponding to Braak stages III-VI [43]), therefore after the detection by tau-PLA. Our data therefore suggest that tau multimerization is an early pathological event in AD.

Although AT8 is the most common tau antibody used for the pathological diagnosis and staging of AD pathology, it shows a preference for late-type hyperphosphorylated tau aggregates [40]. The AT180 marker has been described to detect phosphorylation of tau at Thr321 epitope, one among the earliest PTMs occurring during tau aggregation process [18, 40], which precedes the hyperphosphorylation in the double epitope Ser202/Thr205 recognized by AT8. In addition to this, the MC1 antibody detects early conformational changes of tau. In order to investigate the sequence of events linked to the development of disease regarding multimerization, hyperphosphorylation and misfolding of tau, we performed careful semi-quantitative evaluation of tau-PLA, AT8, AT180 and MC-1 in early Braak stages (0–III). Small-sized stained structures (the diffuse pathology recognized by tau-PLA and dot-/thread-like labelling by tau immunohistochemistry) and larger perikaryal NFT-like structures were quantified separately in temporal isocortex, CA4 sector, entorhinal cortex and CA1 sector.

**Fig. 6:**
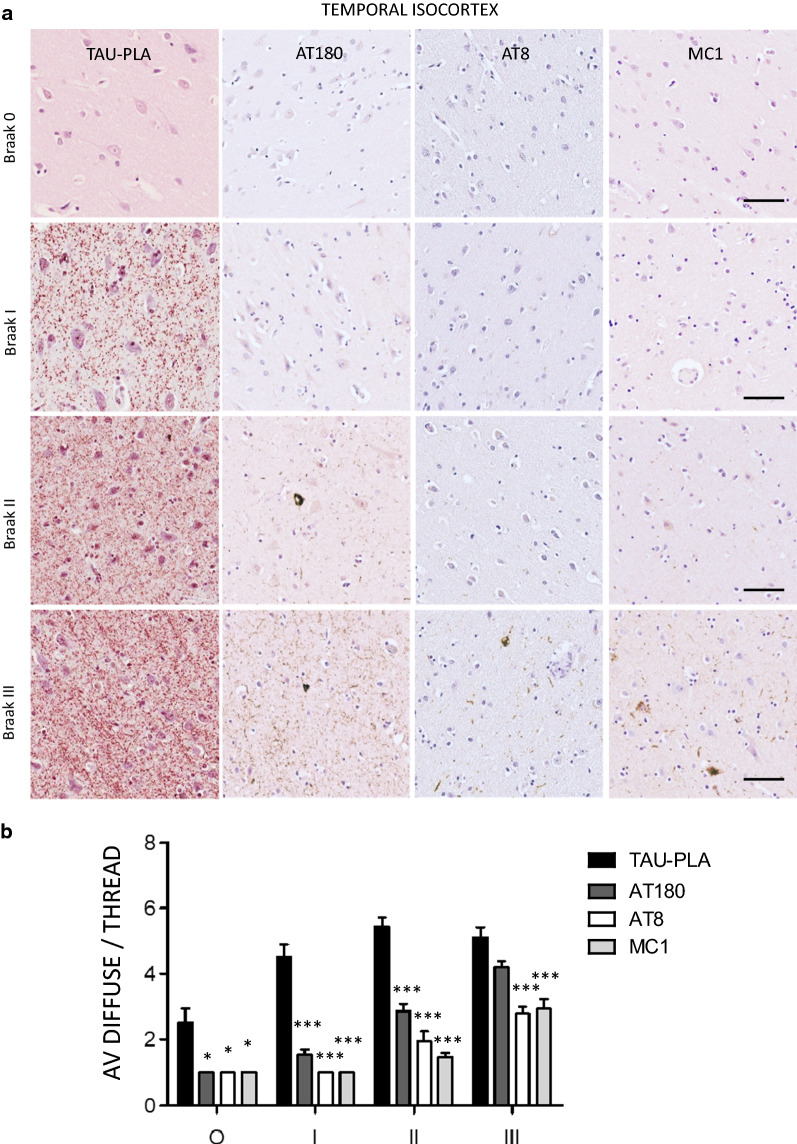
Early tau multimerization detection, prior to detection of tau hyperphosphorylation and misfolding across hippocampal regions and temporal isocortex–temporal isocortex. **a** Representative images of selected hippocampal regions are shown here. FFPE sections of posterior hippocampus at the level of lateral geniculate nucleus from Braak 0 to III were stained with tau-PLA and AT180-, AT8-, MC1-immunohistochemistry. Minimal PLA and immunohistochemistry signal is seen in Braak 0 stage. Tau-PLA reveals abundant pathology in Braak stages I to III, while AT180-immunohistochemistry shows a modest increase in signal in Braak stages I to III. AT8- and MC1-immunohistochemistry is absent or relatively low at initial stages, with signal starting to appear in Braak III. Scale bar 50 μm. **b** Quantification tau-PLA, AT8-, MC1-, and AT8- IHC labelled diffuse pathology. All groups in each Braak stage were compared to tau-PLA through a ONE-WAY ANOVA (Dunnet). N = 11/12/12/9/7/8/8. *p < 0.05, **p < 0.01, ***p < 0.001. *AV* average, *IHC* immunohistochemistry

**Fig. 7:**
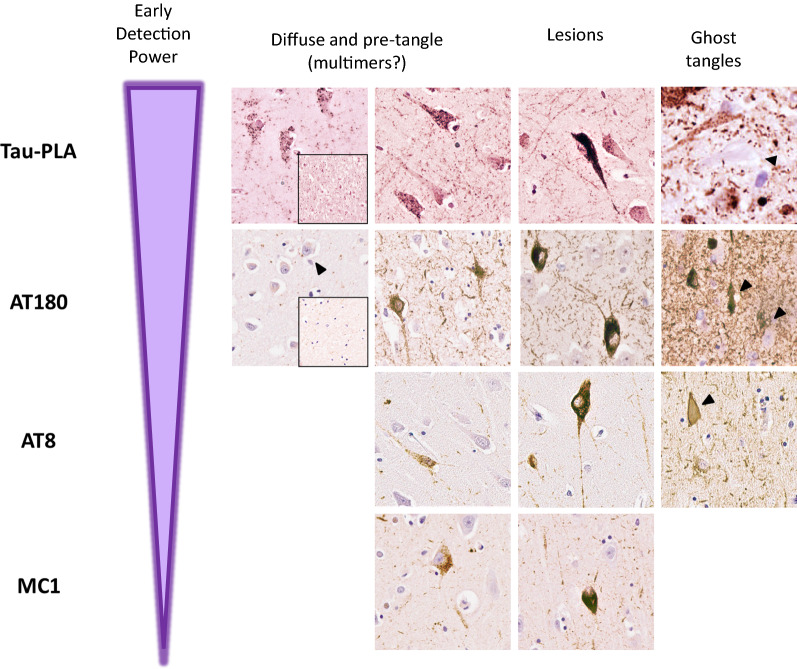
Tau self-interaction as one of the earliest events occurring during neurofibrillary tangle maturity. Schematic representation and images of the dynamic lifespan of NFT. Representative images of the hippocampal region are shown here. FFPE sections of posterior hippocampus at the level of lateral geniculate nucleus, stained with tau-PLA and AT180-, AT8-, MC1-immunohistochemistry. Tau-PLA reveals that multimerization is one of the earliest detectable events occurring during the development of tangles. AT180 positive staining can be detected even before the formation of pretangles, occurring straight after tau self-interaction appears. Hyperphosphorylation recognized by AT8 and conformational changes by MC1 occur early in the maturity of tangles as they are present in both pretangles and mature NFT. Ghost tangles appeared to be positive for AT180 and AT8, but not for MC1 (and possibly tau-PLA)

All three tau antibodies—AT180, AT8 and MC1—were able to detect small-sized structures, in general prior to the detection of larger lesions. However, there was a clear pattern for tau-PLA signal being considerably more abundant than tau-immunohistochemistry in the regions quantified (Fig. 6, Additional file 1: Figs. S8, S9, S10) and to appear earlier. This was particularly evident in temporal isocortex and CA4 sector in Braak stages 0 to II. In these two regions, tau-PLA was the only signal detected in Braak stage I, statistically different from that in Braak 0 (Fig. 6, Additional file 1: Fig. S8). AT180-immunohistochemistry followed tau-PLA in sensitivity, with small-sized structures firstly appearing in Braak stage II in these two regions (Fig. 6, Additional file 1: Fig. S8), their levels approaching those of tau-PLA at stage III in all hippocampal areas except CA4 (Fig. 6, Additional file 1: Figs. S8, S9, S10). These results suggest that AT180 staining can be detected early in the development of fibrillar tangles even before the formation of pretangles, and before AT8 staining, but after tau multimerization starts appearing and being captured by tau-PLA (Fig. 7).

MC1-immunohistochemistry showed a negligible number of MC1 positive small-sized structures or larger lesions until Braak stages II or III, depending of the region (Additional file 1: Fig. S11), at a point when tau-PLA signal is already well-established. This suggests that while the conformational changes of tau recognized by MC1 may occur early in the maturity of tangles, these changes, like phosphorylation, could occur after tau multimerization (Fig. 7). Interestingly, ghost tangles appeared to be positive for AT180 and AT8, but not for MC1 and tau-PLA (Figs. 3, 7).

In the brains used in this study, quantification of lesions labelled with 4G8 antibody showed an overall increase of beta-amyloid load with neurofibrillary stage (Additional file 1: Fig. S12).

Altogether these data support that tau–tau interactions are one of the earliest detectable molecular events occurring during the development of tau pathology, which is especially pronounced in the alveus/stratum oriens.

### The early diffuse pathology detected by tau-PLA is highly sensitive to PK degradation

**Fig. 8:**
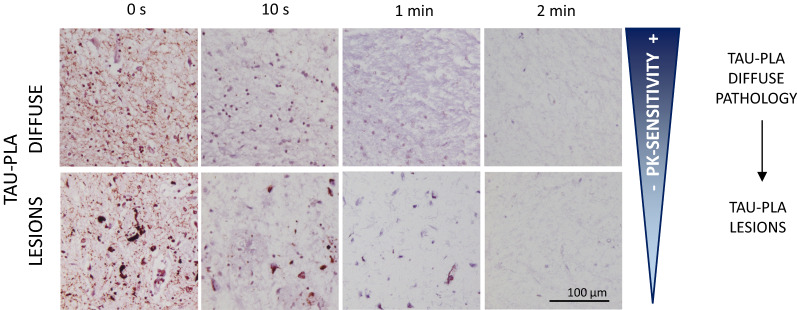
Tau-PLA labelling is very sensitive to PK digestion which has a differential effect on small diffuse complexes and larger neurofibrillary-type lesions. Samples were treated for 0/10/60/120 s with PK at 37 °C and then stained for tau-PLA. Tau-PLA signal was very sensitive to PK treatment. Signal form diffuse small complexes was depleted by 10 s of PK treatment, while larger neurofibrillary-type lesions (neuritic and perikaryal lesions) labelled with tau-PLA required 1 min of PK digestion for complete depletion. NFT: neurofibrillary tangles

The conformational changes associated with the polymerization of amyloid-prone proteins can prevent the access of proteases to their putative cleavage sites [47] and therefore digestion with proteases has been used as a surrogate to infer these conformational changes. In particular, the beta-sheet-rich secondary structure has been closely associated with the typical insolubility and protease resistance of the mature protein aggregates of neurodegenerative diseases [17, 31, 47]. To explore the accessibility of tau5 epitopes within the different tau aggregates labelled by tau-PLA, we performed PK treatment of varying durations (Fig. 8). The labelling of perikaryal lesions by tau-PLA was abolished within 1 min of PK treatment, while the labelling of diffuse pathology by tau-PLA was considerably more sensitive to protease degradation, with signal completely depleted after 10 s of PK digestion. While we cannot rule out that the apparent differences in PK sensitivity could also be due to different overall amounts of multimer or extents of multimer aggregation, as well as interactions of tau with PK-resistant cofactors such as nucleic acids, our results suggest that diffuse pathology is composed most probably of a relaxed structure, very accessible to digestion, whereas tau-PLA signal located in lesions seemed to be of a more compact and PK-resistant nature, which is in agreement with the early nature of diffuse pathology versus that of lesions.

## Discussion

The molecular basis of prion and prion-like disorders is the ability of certain proteins to acquire a self-templating amyloidogenic state, in which protein multimerization is a required step for molecular seeding [12]. Here, we describe tau-PLA, a technique that enables the specific histological visualization in situ of the tau–tau interactions, regardless of tau phosphorylation state and conformational changes, with preservation of the cellular and subcellular morphology. PLA has been used previously to detect both heterotypic [58] as well as homotypic protein interactions [33, 42, 53, 56], which shows the reliability of the PLA approach. We analyzed the presence of tau–tau interactions in FFPE hippocampal and temporal isocortex sections from 67 post-mortem human brains from all Braak stages using tau-PLA and compared with the presence of phosphorylated and misfolded tau using immunohistochemistry. As expected, a range of pathological lesions contain tau multimers. In addition, we identified a previously undetected widespread diffuse pathology that develops ahead and beyond the anatomical confinement of the hyperphosphorylated and misfolded tau material from the earliest Braak stages.

In AD, full-length tau assembles into filaments with cross-beta-amyloid structure [21]. A proportion of tau filaments are typically found abnormally hyperphosphorylated [24, 26]. In vitro, phosphorylation at the Ser202/Thr205/Ser208 sites, together with absence of phosphorylation at the Ser262 site, enables fibrillization [16], and this event is recognized by AT8 marker [41]. AT8-positive pathology has been recognized in dystrophic neurites and granular perikaryal tau deposits in pre-tangle neurons [5, 59], constituting an early phosphoepitope during cellular tau pathology development. Similarly, AT180 detects tau phosphorylated at Th231, a phosphoepitope affected early in the development of tau pathology and connected with tau multimerization in AD brain [18]. Tau protein also exhibits conformational changes [10, 32]. Misfolding recognized by MC1 antibody, where the N-terminal and the third microtubule binding domain of tau are in close proximity, has been described as one of the earliest PTMs of tau in AD [65]. However, phosphorylation and misfolding are not a requirement for the in vitro assembly of full-length tau [22, 23, 27, 34, 48, 52, 68, 69] while seed propagation of tau can happen independently of phosphorylation [20]. Therefore, the visualization of the key molecular event of tau multimerization could undercover a breath of pathological tau forms occurring ahead or independently of tau hyperphosphorylation and misfolding, which our tau-PLA data support. The data obtained through tau-PLA analysis would be consistent with the maximal distance between the two epitopes in the PLA assay being ~ 16 nm, only slightly larger than the distance required for resonance energy transfer between fluorophores (~ 10 nm) [61], only achieved upon true molecule interaction.

In physiological conditions in human brain, the majority of tau appears to be unaltered and in monomeric state, as bona fide Braak 0 hippocampi showed absent or minimal AT8, AT180, MC1, and tau-PLA signal, on average. Tau-PLA revealed that cases in Braak 0 group presented heterogenicity, indicating that in a subgroup of asymptomatic individuals with no tau pathology defined by immunohistochemistry, tau multimerization has already started. Further study is nevertheless required to determine if the small amount of signal in some Braak 0 cases corresponds to early pathology or a physiological role for tau multimers—a role for tau oligomerization has been suggested in cultured cells [66].

From Braak I stage there is a rise in tau pathology, in most areas studied heralded by small-sized diffuse tau-PLA structures negative for other markers, with AT180-immunohistochemistry being the only marker able to significantly detect, although with lower sensitivity, small-sized tau pathology at the earliest stages. Hyperphosphorylated or misfolded tau recognized by immunohistochemistry was scarce and neuroanatomically confined in early stages; as expected, these lesions were also labelled by tau-PLA, not surprising as they are formed of tau–tau interactions [38]. The absence of AT8-, AT180-, and MC1-immunohistochemistry signal in most of the areas labelled with tau-PLA in the earliest stages suggests that the small-sized diffuse pathology could be composed of non-phosphorylated or non-misfolded tau. Therefore, our data suggest both at the anatomical and cellular level, that tau multimerization is one of the earliest events occurring during the development of AD tau pathology in the brain. Our work parallels recent studies that found that tau multimerization with seeding potential anticipated phospho-tau pathology and suggests the possibility of exploring tau-PLA as a morphologically preserved surrogate of seeding activity [28, 35, 37]. Additional recent work also supports our claim that there is extensive tau multimerization at early stages [18]. However, Ercan-Herbst et al. suggested that a signature of phosphorylated sites which included Thr231 (at least in the entorhinal cortex) was concomitant with multimerization. Unlike ours, this study pooled Braak 0-Braak I cases as control, rather than separated groups, limiting the resolution in the determination of sequence of events at this important transition. In addition, their multimerization assay also lacks the morphological preservation and spatial resolution provided by tau-PLA [18, 28, 37].

Staining of the alveus/stratum oriens and the white matter by tau-PLA and alignment onto axonal morphologies were prominent at early AD stages, as well as at the striatal pencil fibers in P301S mice, suggesting—unsurprisingly given tau functions to stabilize axonal microtubules—this early trigger may occur in axons [15, 29]. Although the majority of small-sized diffuse multimers were observed in neurons, a proportion of them was also localized to microglia, astrocytes and oligodendrocytes from early on, suggesting a potential role to lesion progression, spread and toxicity. It is also interesting that at later stages, the amount of diffuse tau-PLA decreased. This may be due to higher number, at later stages, of ghost tangles, which do not appear labelled by tau-PLA, but also to the possibility that NFTs could be a sink for earlier pathological species [11, 39], which disappear in the maturation process of the tangle. The epitope recognized by the antibody we used to build the tau-PLA assay, tau5, maps to aminoacids 218–225 [51] which are part of the fuzzy coat of tau PHFs and SFs. The fuzzy coat is largely lost in extracellular ghost tangles [21], which would also explain the lack of labelling of ghost tangles by tau-PLA. The neuronal loss in AD is far superior to the amount of NFTs [25], which points to an unknown intermediate as a neurotoxic agent and point of therapeutic intervention. While more investigation is required to interrogate whether the early tau multimerization detected by tau-PLA could be playing a role as these toxic intermediates, our data using PK digestion suggests the small diffuse multimers have a distinct conformation, different from the bulk of neurofibrillary tau.

As mentioned, a region of tau corresponding to part of the repeats forms a protease resistant core when assembled into filaments [21]. Exposure of this core normally requires prolonged protease treatment [46, 60, 70]. In agreement with this idea, complete depletion of large lesions labelled with tau-PLA required at least 1 min of PK treatment, while in contrast, labelling of the small diffuse pathology by tau-PLA was completely abolished after only 10 s of digestion. This does not preclude that these species may contain a component of beta-sheet secondary structure centered in the region of the repeats but indicates that the overall assembly of the structure seems to allow full and easy access to the tau5 epitope and is tempting to speculate that they represent early oligomeric species like those thought to be toxic [19]. We cannot rule out that the apparent differences in PK sensitivity could also be due to different amounts of multimer or extents of multimer aggregation, e.g. bundling or clustering of aggregates, rather than differences in secondary, tertiary or quaternary structures of tau molecules within individual aggregates. Another possible explanation is that an increased association of PK-resistant cofactors (such as nucleic acids) in lesions would also confer resistance to PK treatment.

We must also recognize that inferring a temporal sequence of events from post-mortem samples has its limitations and does not establish causality. At this point, we cannot categorically affirm if this diffuse pathology actually is or converts into hyperphosphorylated and misfolded neurofibrillary material, as alternative explanations are theoretically possible. For example, diffuse tau-PLA could be a marker of early cellular alterations responsible for the posterior tau neurofibrillary aggregation. Neither can we exclude that small multimers may diffuse from localized large lesions. If that was the case, however, we would expect a gradient of tau-PLA, with highest levels in areas close or connected to those harboring immunohistochemistry-positive lesions, but the opposite is indeed the case. Lastly, it could be hypothesized that tau diffuse pathology is a normal part of aging. The detailed neuroanatomical analysis of pre-tangle Braak stages, i.e. the early subcortical stages described by Braak [8], and further experimental work with cellular and animal models could help to definitively establish the sequence of events: what is the toxicity and seeding potential of these small diffuse multimers, their contribution to neuronal dysfunction, spread of pathology and cell death as well as the role of glial cells.

Regardless of these unanswered questions, our tool can play an important role in dissecting the pathophysiology of early aggregates, in the design of early diagnostic methods and the monitoring of efficacy of treatments. Our data also challenges therapeutic approaches focused solely on modulating tau phosphorylation and misfolding. However, given the breath of studies indicating AD is a prion-like disorder, our findings here of an early and widespread tau multimerization could mark a crucial early transition point, at which an intervention may provide a disease-modifying therapy.

## Conclusion

Here, we have developed for the first time a novel assay, tau-PLA, that allows the specific detection of tau–tau interactions, without monomeric recognition, both in vitro and in situ. In addition to staining neurofibrillary-like lesions in human brain and in contrast to standard histopathological markers, tau-PLA can recognize an early and previously unreported type of diffuse pathology. In this study, we show that tau multimerization is an early molecular event in the development of AD tau pathology in the brain.

## Supplementary Information

**Additional file 1. Fig. S1.** Automated analysis and semi-quantitative scales used for analysis. A and B) Original images and images after analysis with quantification. Analysis performed as specified in Materials and methods. A) 3 sample images with low, middle and high density of diffuse small tau-PLA labelled structures were processed with ImageJ for the automated measurement of diffuse signal, insets show final counts. B) Samples from the 4 different immunolabellings were processed with ImageJ for the automated measurement of large labelled lesions, insets show final counts. C) Semi-quantitative scale used for semi-quantitative analysis of all brain regions. **Fig. S2.** Detection of tau–tau interactions in HEK 293 cells. Representative fields of view of the tau-PLA puncta quantification of Fig. 2B. Tau-PLA puncta are in red. Nuclei were identified by DAPI staining (blue). Scale bar: 10 μm. **Fig. S3.** In situ specificity of tau-PLA in mouse brain tissue. Top: Representative images from 6 months-old P301S transgenic, C57BL/6 wild-type and *MAPT* KO mice (N = 6 per genotype), stained with Tau-PLA and AT8. Brain histological analysis revealed an absence of tau-PLA signal in *MAPT* KO mouse brain tissue. C57BL/6 control mice showed almost negligible load of tau-PLA, whereas this signal appeared to be quite strong in the P301S mice. Tau-PLA revealed prominent tau multimerization in the CA1 region of hippocampus and striatum, anatomical areas negative for AT8. Bottom: Representative images from P301S transgenic animals at the age of 3 (N = 2), 6 (N = 6), and 9 (N = 2) months old. This analysis shows an age-dependent accumulation of tau-PLA signal. Scale bar 100 μm. **Fig. S4.** Tau-PLA detects endogenous human tau–tau interaction in situ. A) Tau-PLA recognized tau pathology in the CA4/dentate gyrus of Alzheimer’s disease (Braak V) as compared to Braak 0 controls. B) Tau-PLA was performed in samples of different Braak stages in the presence or absence of ligase. The absence of ligase in the PLA reaction resulted in the ablation of all PLA signal, indicating the staining is dependent on the ligation of two conjugates. C) 6xHisTag IHC and 6xHisTag-PLA performed in samples of Braak stage 0 and V. No signal was detected after performing immunohistochemistry with 6xHisTag antibody and 6xHisTag-PLA. Scale bar 50 μm. **Fig. S5.** A proportion of diffuse tau complexes locates to astrocytes and microglia. Representative co-immunofluorescence images (Braak IV) of indicated markers and tau-PLA. Arrowheads indicate astrocyte/microglial cells in top and bottom panels, respectively. **Fig S6.** Tau-PLA revealed that cases in Braak 0 group presented heterogenicity. A) Quantification of tau-PLA labelled small diffuse complexes in the different hippocampal regions, revealing the distribution of the population in Braak 0 group. ONE-WAY ANOVA (Tukey). AV; average. B) Representative images of EC stained with tau-PLA that illustrate the heterogenicity that characterize the Braak 0 group. Scale bar 50 μm. **Fig. S7.** Quantification of lesions in samples labelled with tau5 IHC. Samples immunolabelled with tau5 IHC were used for automated analysis. All groups were compared to control (Braak 0) through a ONE-WAY ANOVA (Dunnet). N = 7/7/7/4/6/6/6. AV; average. IHC: immunohistochemistry. **Fig. S8.** Early tau multimerization detection, prior to detection of tau hyperphosphorylation and misfolding across hippocampal regions and temporal isocortex – CA4. A) Representative images of selected hippocampal regions are shown here. FFPE sections of posterior hippocampus at the level of lateral geniculate nucleus from Braak 0 to III were stained with tau-PLA and AT180-, AT8-, MC1-immunohistochemistry. Minimal PLA and immunohistochemistry signal is seen in Braak 0. Tau-PLA reveals abundant pathology in Braak stages I to III, while AT180-immunohistochemistry shows a modest increase in signal in Braak stages I to III. AT8- and MC1-immunohistochemistry is absent or relatively low at initial stages. Scale bar 50 μm. B) Quantification of tau-PLA, AT8-, MC1-, and AT8- IHC labelled diffuse/thread pathology. All groups in each Braak stage were compared to tau-PLA through a ONE-WAY ANOVA (Dunnet). N = 11/12/12/9/7/8/8. AV; average. IHC: immunohistochemistry. **Fig. S9.** Early tau multimerization detection, prior to detection of tau hyperphosphorylation and misfolding across hippocampal regions and temporal isocortex – Entorhinal Cortex. A) Representative images of selected hippocampal regions are shown here. FFPE sections of posterior hippocampus at the level of lateral geniculate nucleus from Braak 0 to III were stained with tau-PLA and AT180-, AT8-, MC1-immunohistochemistry. Minimal PLA and immunohistochemistry signal is seen in Braak 0. Tau-PLA reveals abundant pathology in Braak stages I to III, while AT180-immunohistochemistry shows a modest increase in signal in Braak stages I to III. AT8- and MC1-immunohistochemistry is absent or relatively low at initial stages, with signal appearing in Braak III. Scale bar 50 μm. B) Quantification tau-PLA, AT8-, MC1-, and AT8- IHC labelled diffuse pathology. All groups in each Braak stage were compared to tau-PLA through a ONE-WAY ANOVA (Dunnet). N = 11/12/12/9/7/8/8. AV; average. IHC: immunohistochemistry. **Fig. S10.** Early tau multimerization detection, prior to detection of tau hyperphosphorylation and misfolding across hippocampal regions and temporal isocortex – CA1. A) Representative images of selected hippocampal regions are shown here. FFPE sections of posterior hippocampus at the level of lateral geniculate nucleus from Braak 0 to III were stained with tau-PLA and AT180-, AT8-, MC1-immunohistochemistry. Minimal PLA and immunohistochemistry signal is seen in Braak 0. Tau-PLA reveals abundant pathology in Braak stages I to III, while AT180-immunohistochemistry shows a modest increase in signal in Braak stages I to III. AT8- and MC1-immunohistochemistry is absent or relatively low at initial stages, with signal appearing in Braak III. Scale bar 50 μm. B) Quantification tau-PLA, AT8-, MC1-, and AT8- IHC labelled diffuse pathology. All groups in each Braak stage were compared to tau-PLA through a ONE-WAY ANOVA (Dunnet). N = 11/12/12/9/7/8/8. AV; average. IHC: immunohistochemistry. **Fig. S11.** Quantification of diffuse pathology/threads and lesions labelled by AT180- and MC1- IHC in hippocampal regions and temporal isocortex. Quantification of diffuse pathology/threads and lesions labelled by AT180- and MC1- IHC in hippocampal regions and temporal isocortex. All groups were compared to control (Braak 0) through a ONE-WAY ANOVA (Dunnet). N = 11/12/12/9. AV; average. IHC: immunohistochemistry. **Fig. S12.** Quantification of lesions in samples labelled with 4G8 IHC. Clinically processed samples immunolabelled with 4G8 were used for automated analysis. All groups were compared to control (Braak 0) through a ONE-WAY ANOVA (Dunnet). N = 7/6/6/4/5/5/4. AV; average. IHC: immunohistochemistry. **Table S1.** Summary of patient information. AV; average. **Table S2.** Additional information of brain samples. PMI: post-mortem interval, CERAD: consortium to establish a registry for Alzheimer’s disease, MCI: mild cognitive impairment.

## Data Availability

All data generated or analysed during this study are included in this published article and supplementary files.

## Abbreviations

BiFC: Bimolecular fluorescence complementation
ELISA: Enzyme-linked immunosorbent assay
FFPE: Formalin-Fixed Paraffin-Embedded
NFTs: Neurofibrillary tangles
NT: Neuropil thread
PHFs: Paired helical filaments
PFA: Paraformaldehyde
PBS: Phosphate-buffered saline
PK: Proteinase K
PLA: Proximity ligation assay
PTM: Post-translational modification
RT: Room temperature
SPs: Senile plaques
SF: Straight filaments
Tau-PLA: Tau proximity ligation assay
TEM: Transmission electron microscopy
WB: Western blot
