# Supplementary material for: Tau-proximity ligation assay reveals extensive previously undetected pathology prior to neurofibrillary tangles in preclinical Alzheimer’s disease
Source: Acta Neuropathol Commun. 2021 Jan 28;9:18. doi: 10.1186/s40478-020-01117-y (PMC7844979; doi:10.1186/s40478-020-01117-y)
Supplement: Supplementary file 1 — Additional file 1. Fig. S1. Automated analysis and semi-quantitative scales used for analysis. A and B) Original images and images after analysis with quantification. Analysis performed as specified in Materials and methods. A) 3 sample images with low, middle and high density of diffuse small tau-PLA labelled structures were processed with ImageJ for the automated measurement of diffuse signal, insets show final counts. B) Samples from the 4 different immunolabellings were processed with ImageJ for the automated measurement of large labelled lesions, insets show final counts. C) Semi-quantitative scale used for semi-quantitative analysis of all brain regions. Fig. S2. Detection of tau–tau interactions in HEK 293 cells. Representative fields of view of the tau-PLA puncta quantification of Fig. 2B. Tau-PLA puncta are in red. Nuclei were identified by DAPI staining (blue). Scale bar: 10 μm. Fig. S3. In situ specificity of tau-PLA in mouse brain tissue. Top: Representative images from 6 months-old P301S transgenic, C57BL/6 wild-type and MAPT KO mice (N = 6 per genotype), stained with Tau-PLA and AT8. Brain histological analysis revealed an absence of tau-PLA signal in MAPT KO mouse brain tissue. C57BL/6 control mice showed almost negligible load of tau-PLA, whereas this signal appeared to be quite strong in the P301S mice. Tau-PLA revealed prominent tau multimerization in the CA1 region of hippocampus and striatum, anatomical areas negative for AT8. Bottom: Representative images from P301S transgenic animals at the age of 3 (N = 2), 6 (N = 6), and 9 (N = 2) months old. This analysis shows an age-dependent accumulation of tau-PLA signal. Scale bar 100 μm. Fig. S4. Tau-PLA detects endogenous human tau–tau interaction in situ. A) Tau-PLA recognized tau pathology in the CA4/dentate gyrus of Alzheimer’s disease (Braak V) as compared to Braak 0 controls. B) Tau-PLA was performed in samples of different Braak stages in the presence or absence of ligase. The absence of ligase [file 40478_2020_1117_MOESM1_ESM.pdf]

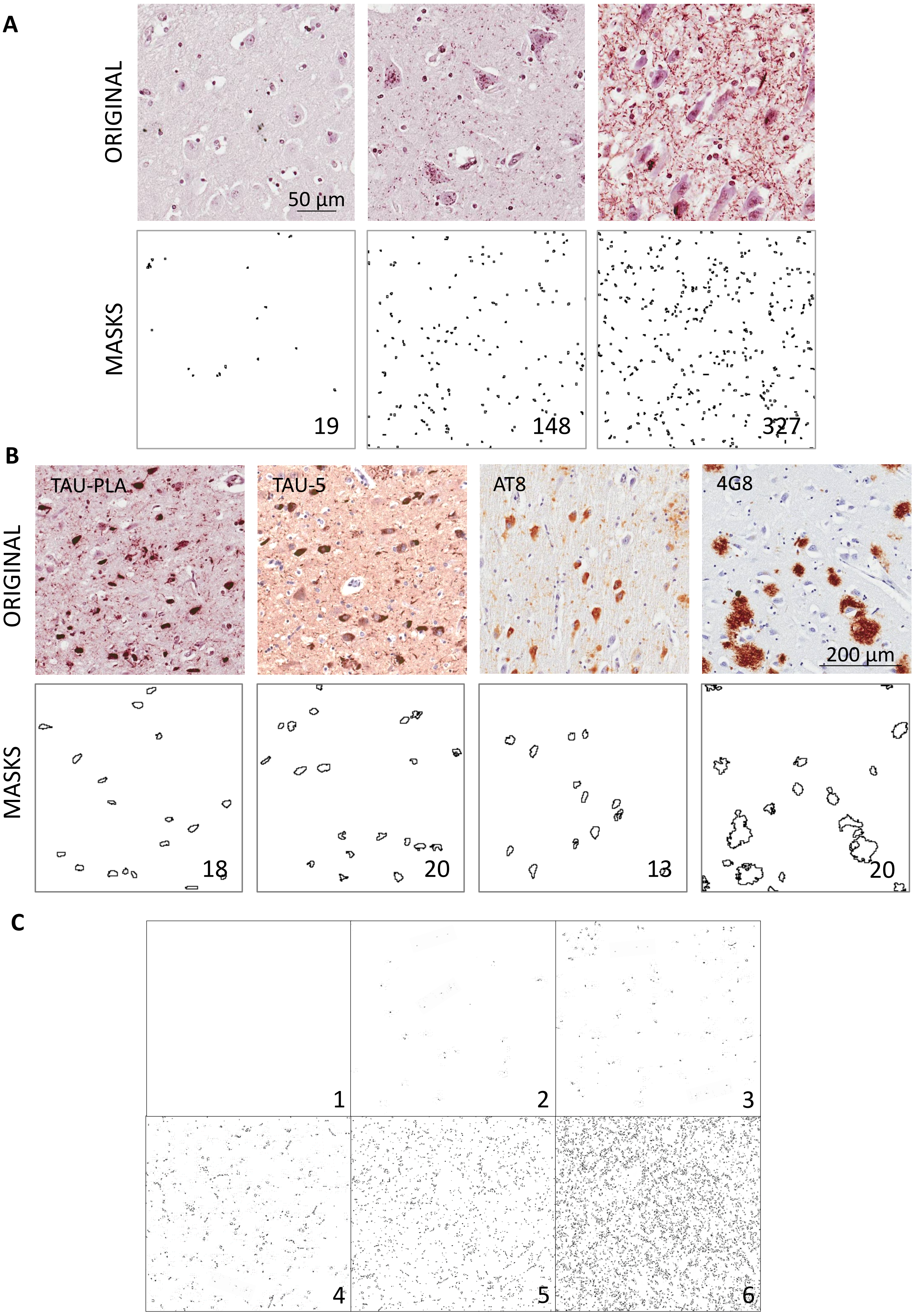

Figure S1. Automated analysis and semi-quantitative scales used for analysis.

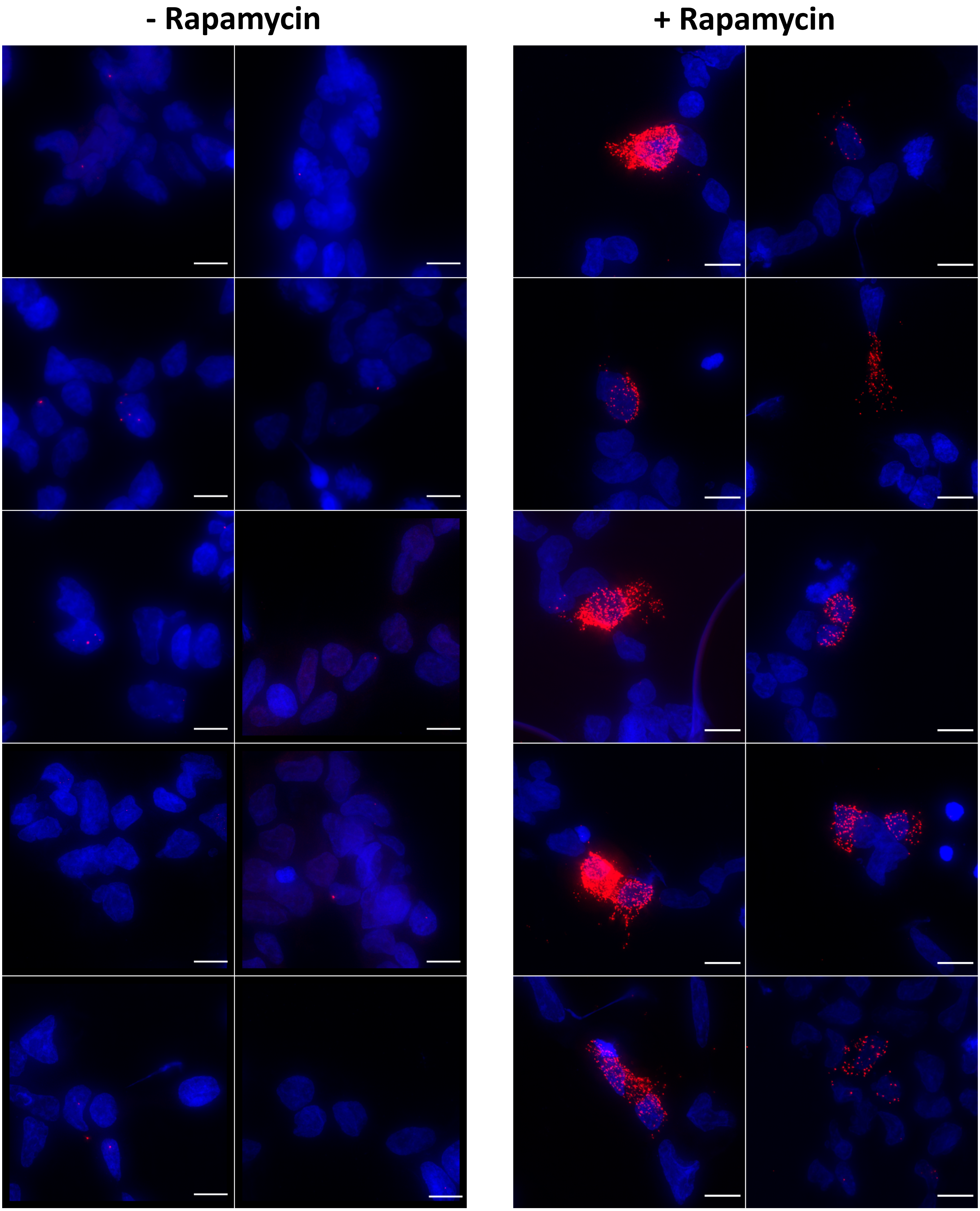

Figure S2. Detection of tau-tau interactions in HEK293 cells.

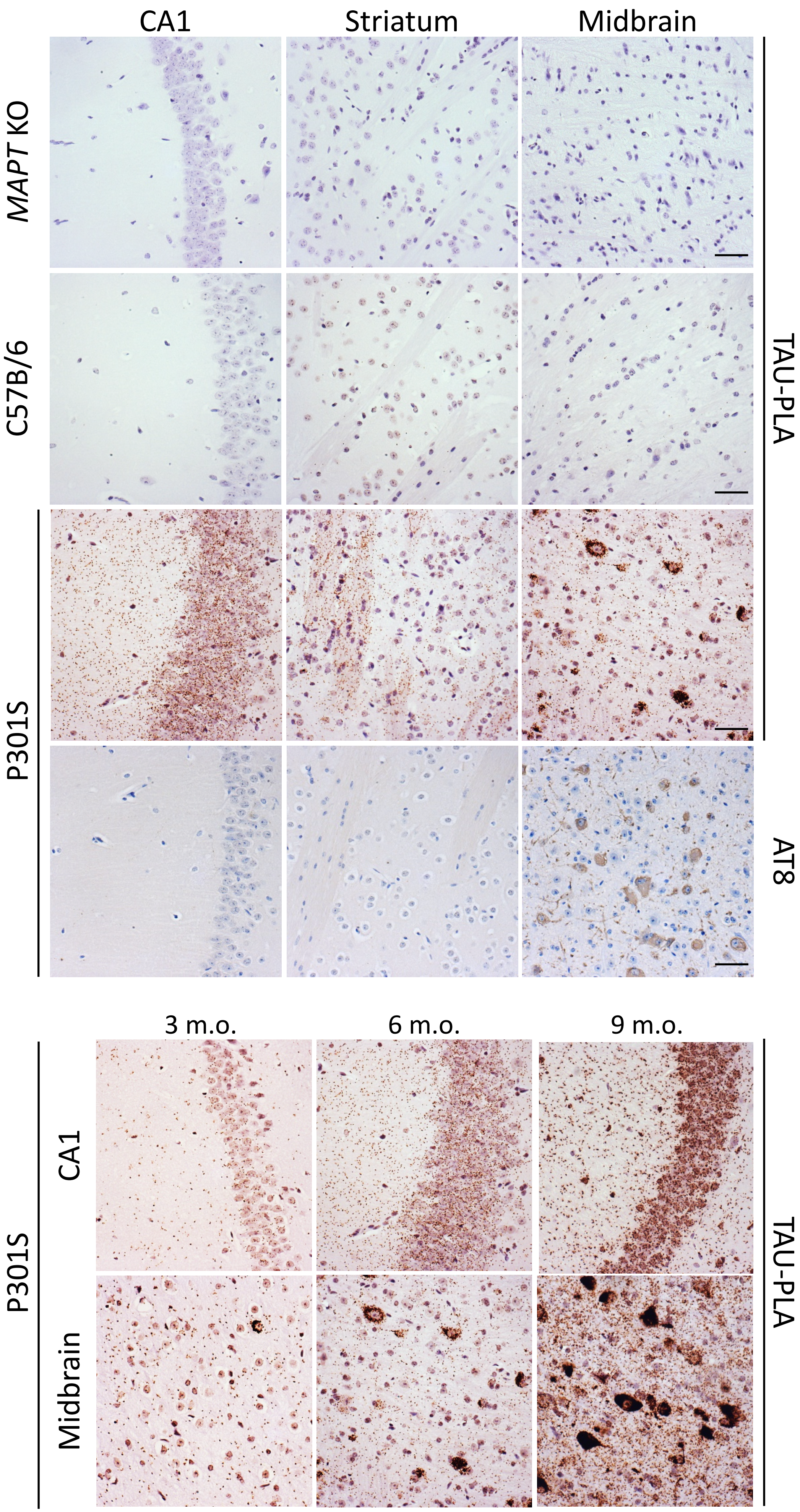

Figure S3. Demonstration of the *in situ* specificity of tau-PLA in mouse brain tissue.

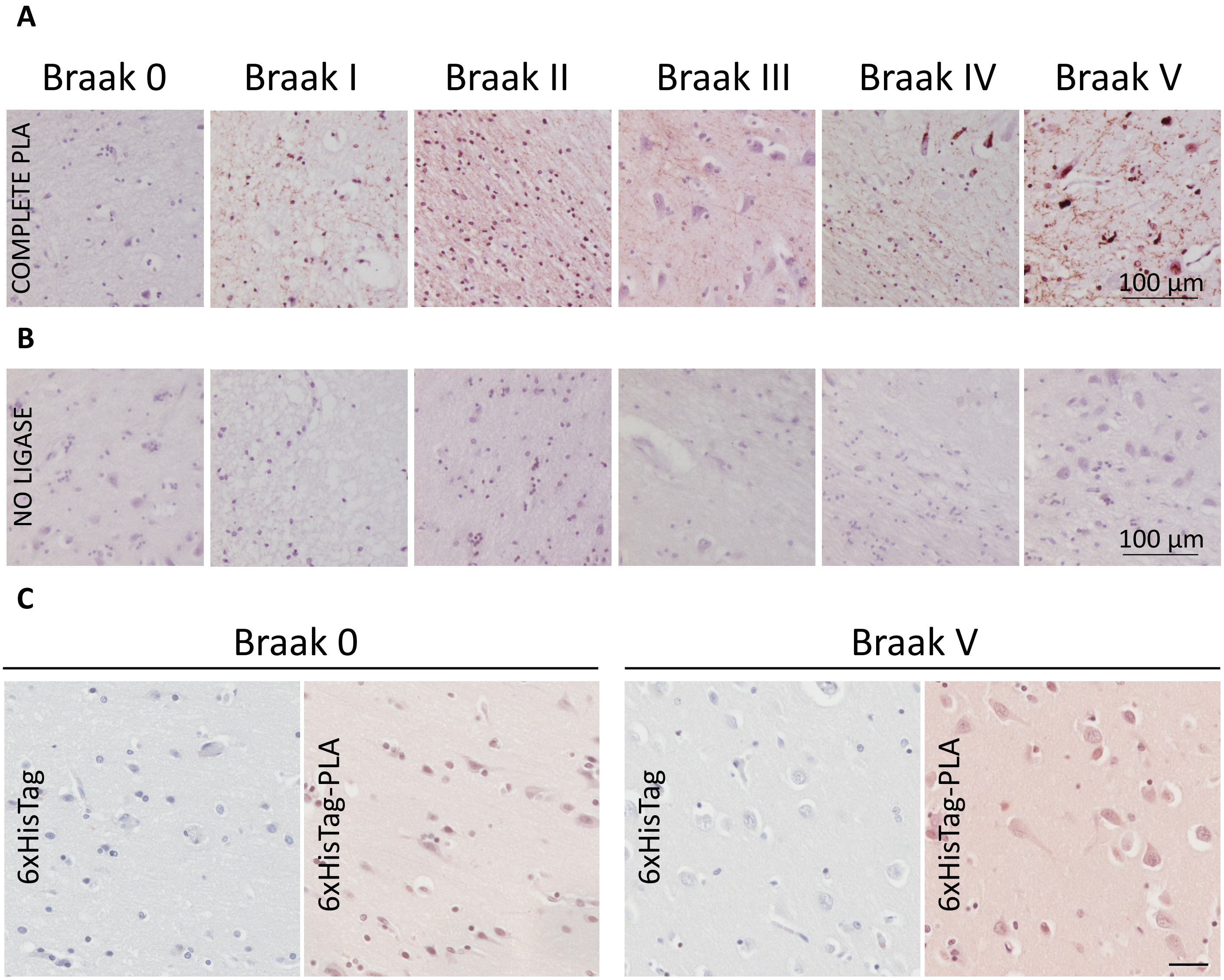

Figure S4. Tau-PLA detects endogenous human tau-tau interaction *in situ*.

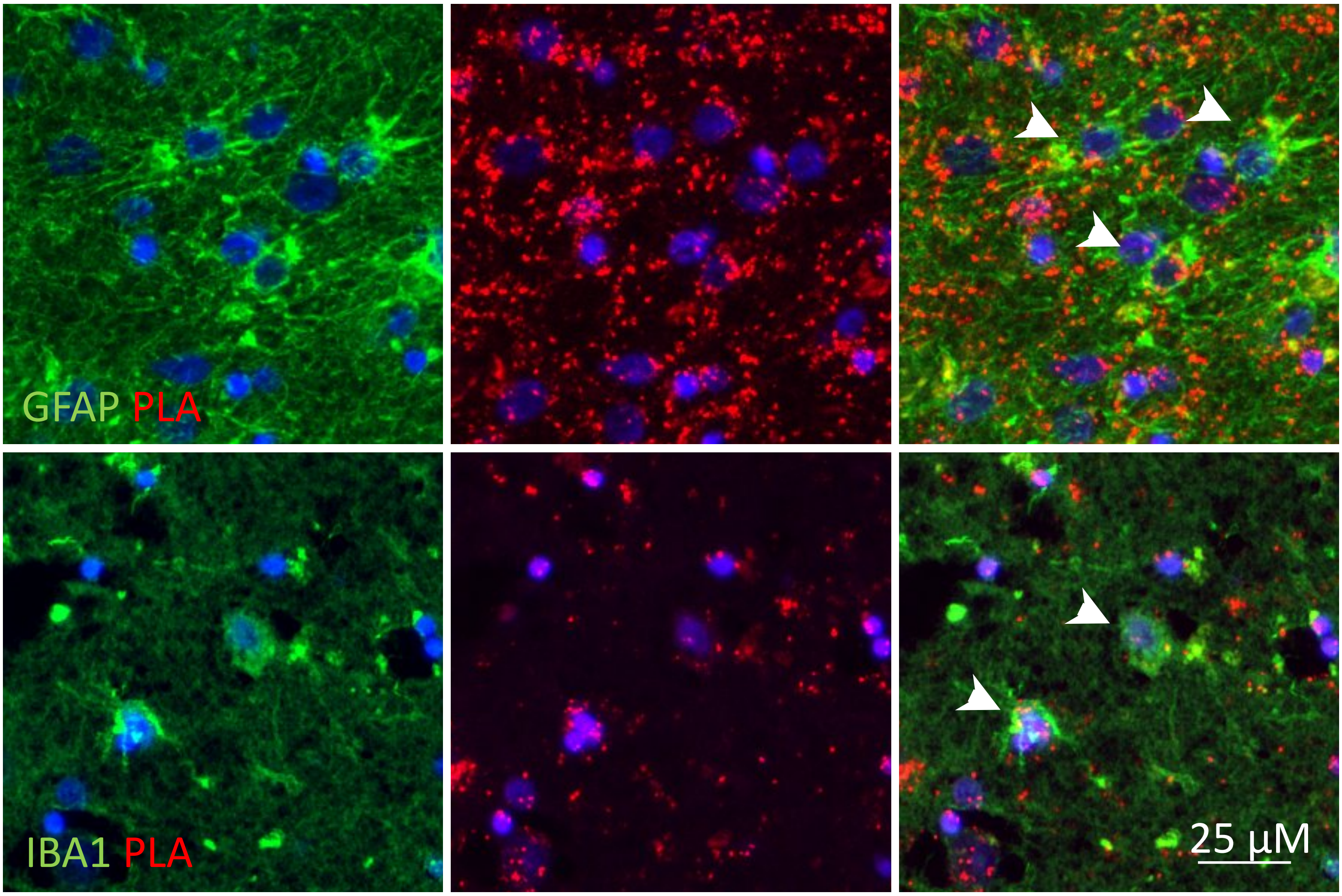

Figure S5. A proportion of diffuse tau complexes locates to astrocytes and microglia.

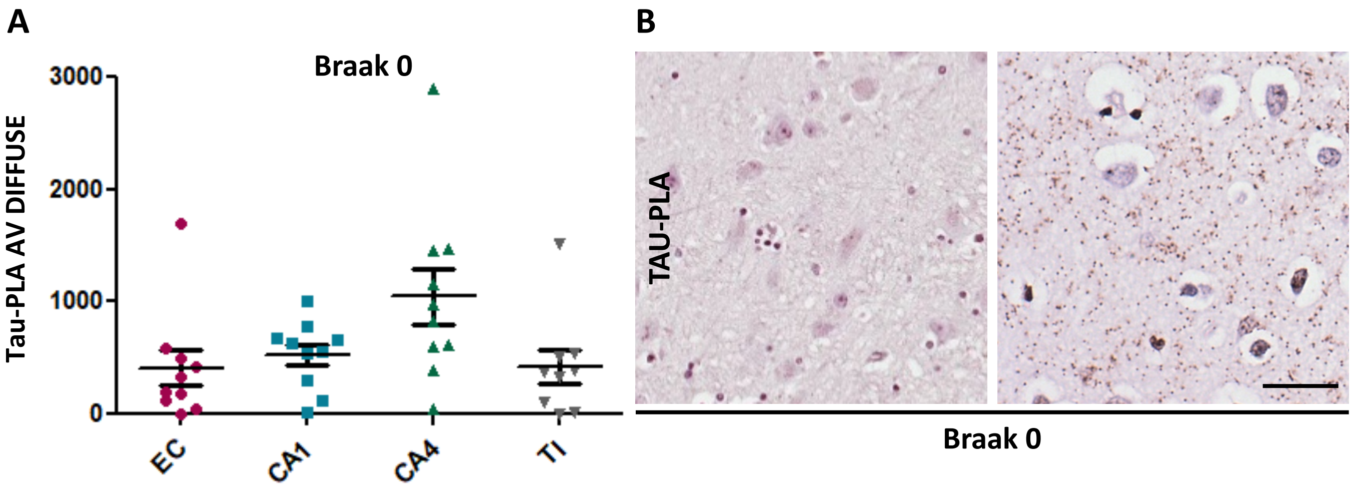

Figure S6. Tau-PLA revealed that cases in Braak 0 group presented heterogenicity.

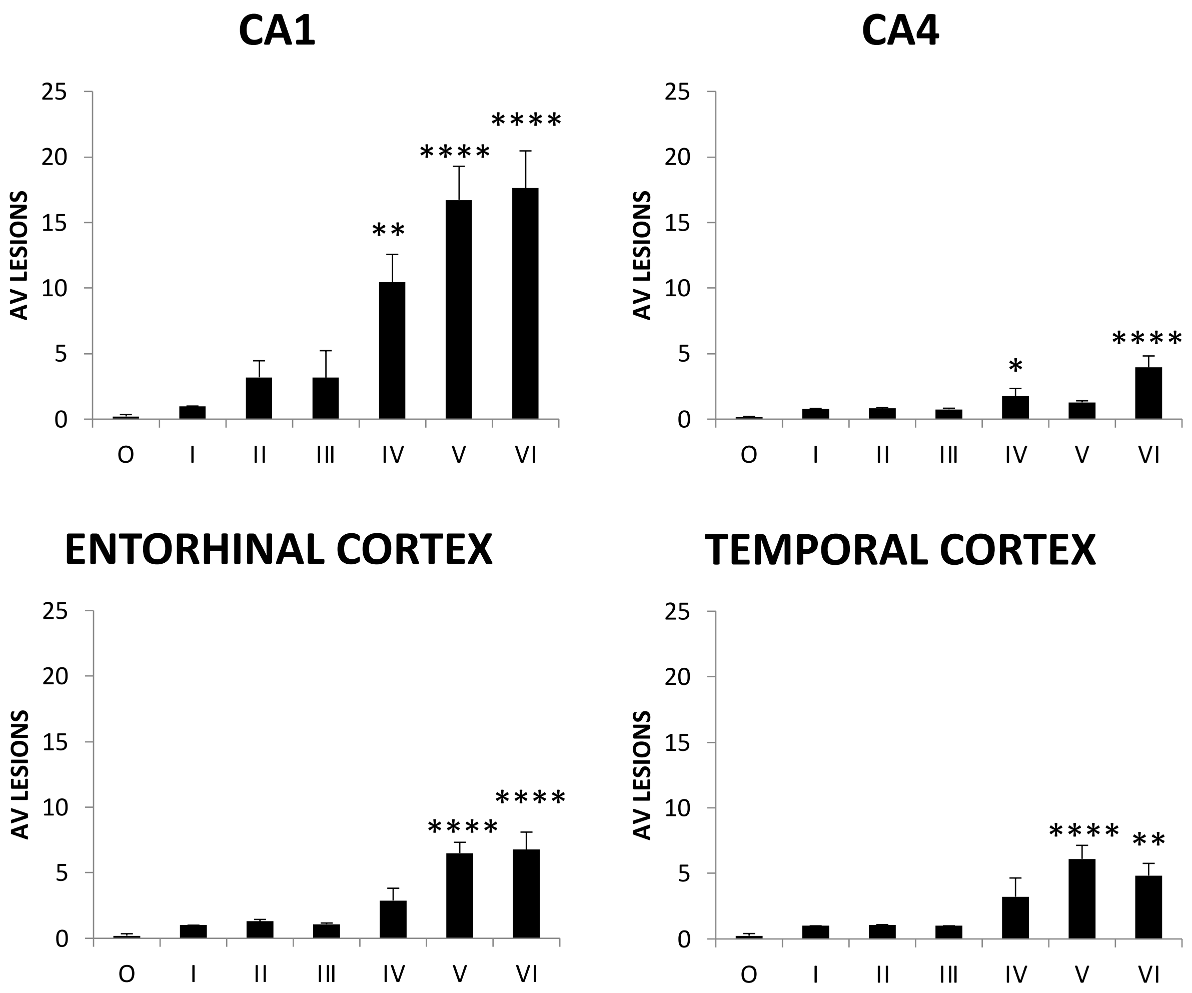

Figure S7. Quantification of lesions in samples labelled with tau5 IHC.

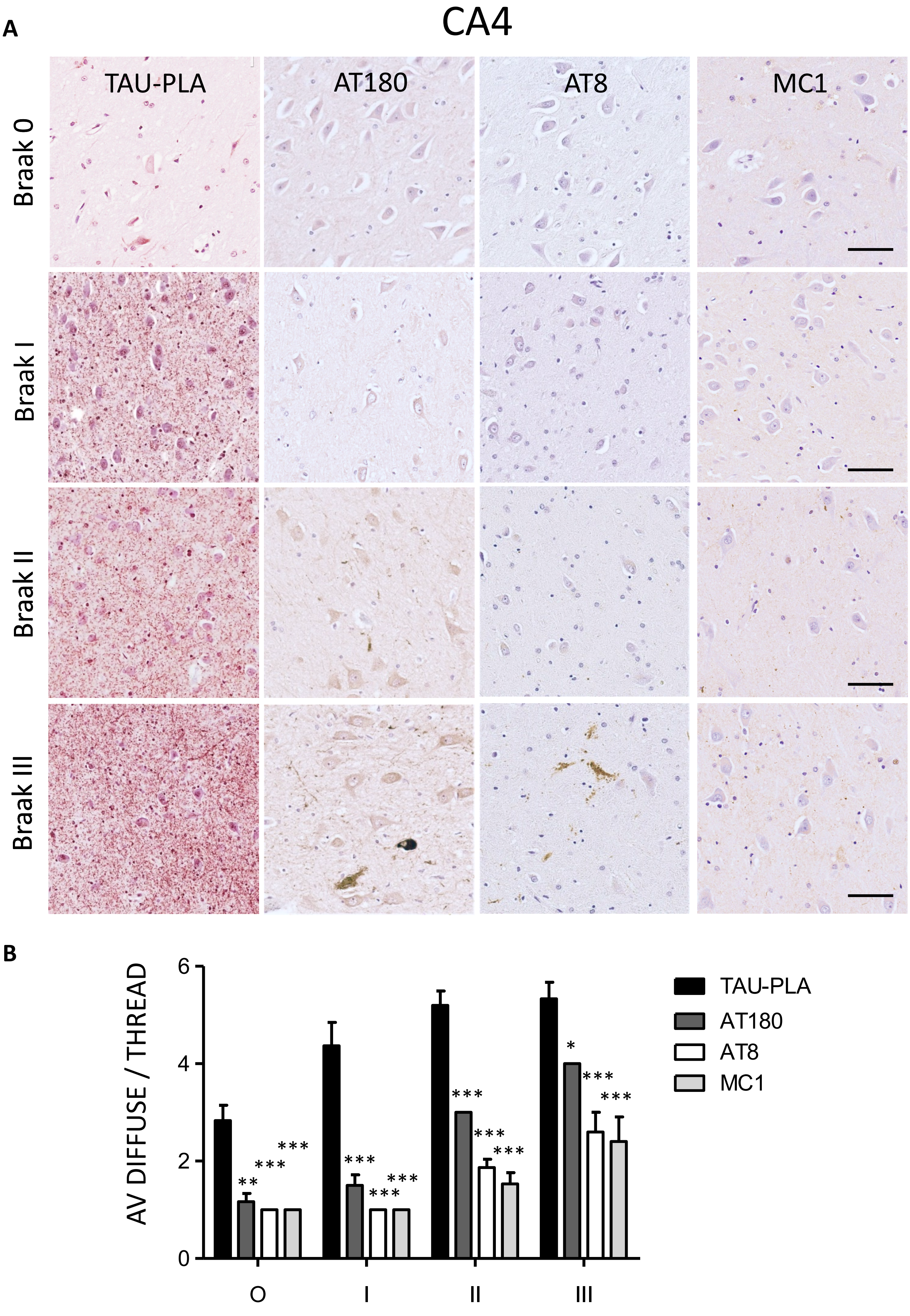

Figure S8. Early tau multimerization detection, prior to detection of tau hyperphosphorylation and misfolding across hippocampal regions and temporal isocortex– CA4.

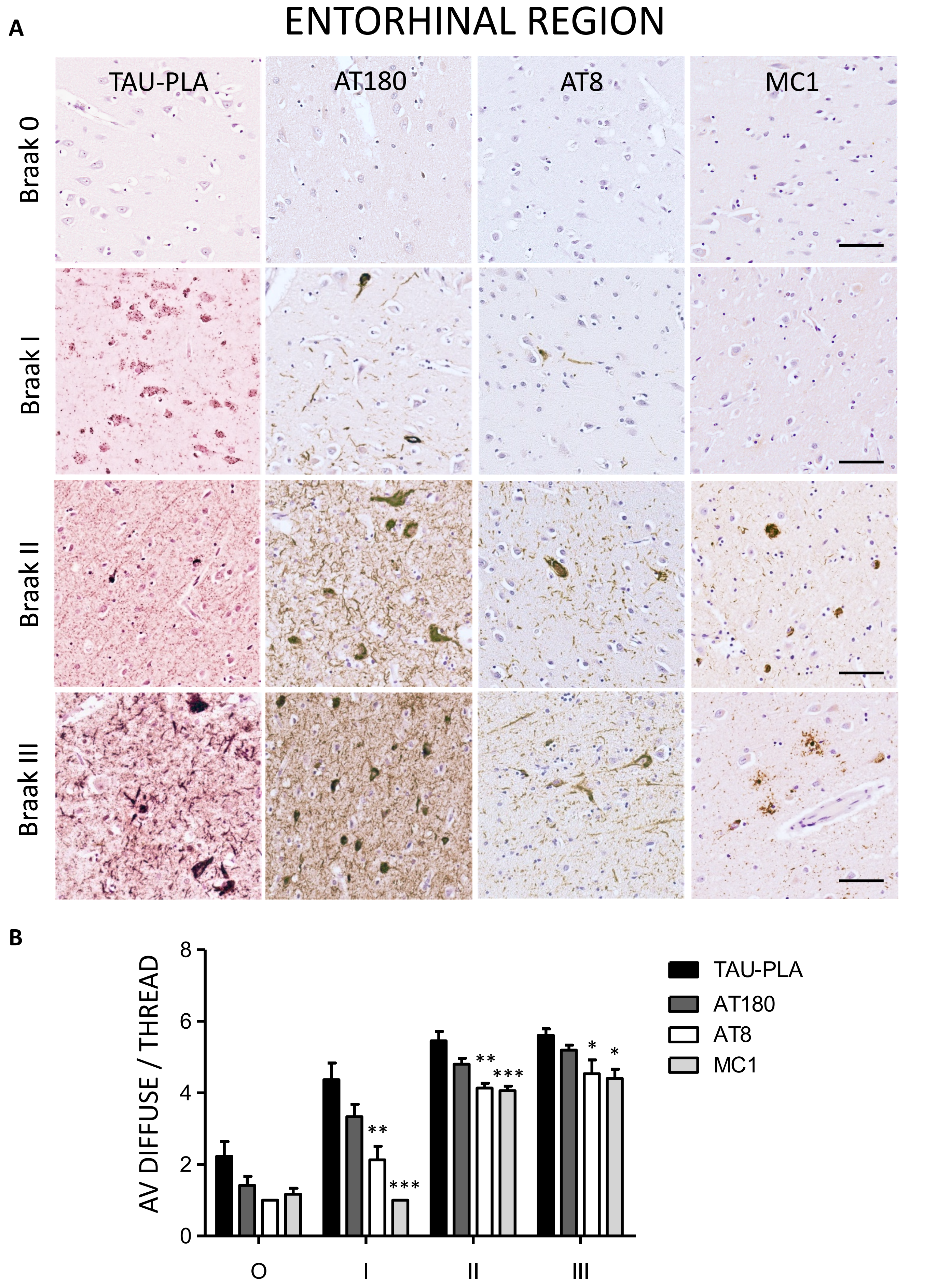

Figure S9. Early tau multimerization detection, prior to detection of tau hyperphosphorylation and misfolding across hippocampal regions and temporal isocortex– Entorhinal Cortex.

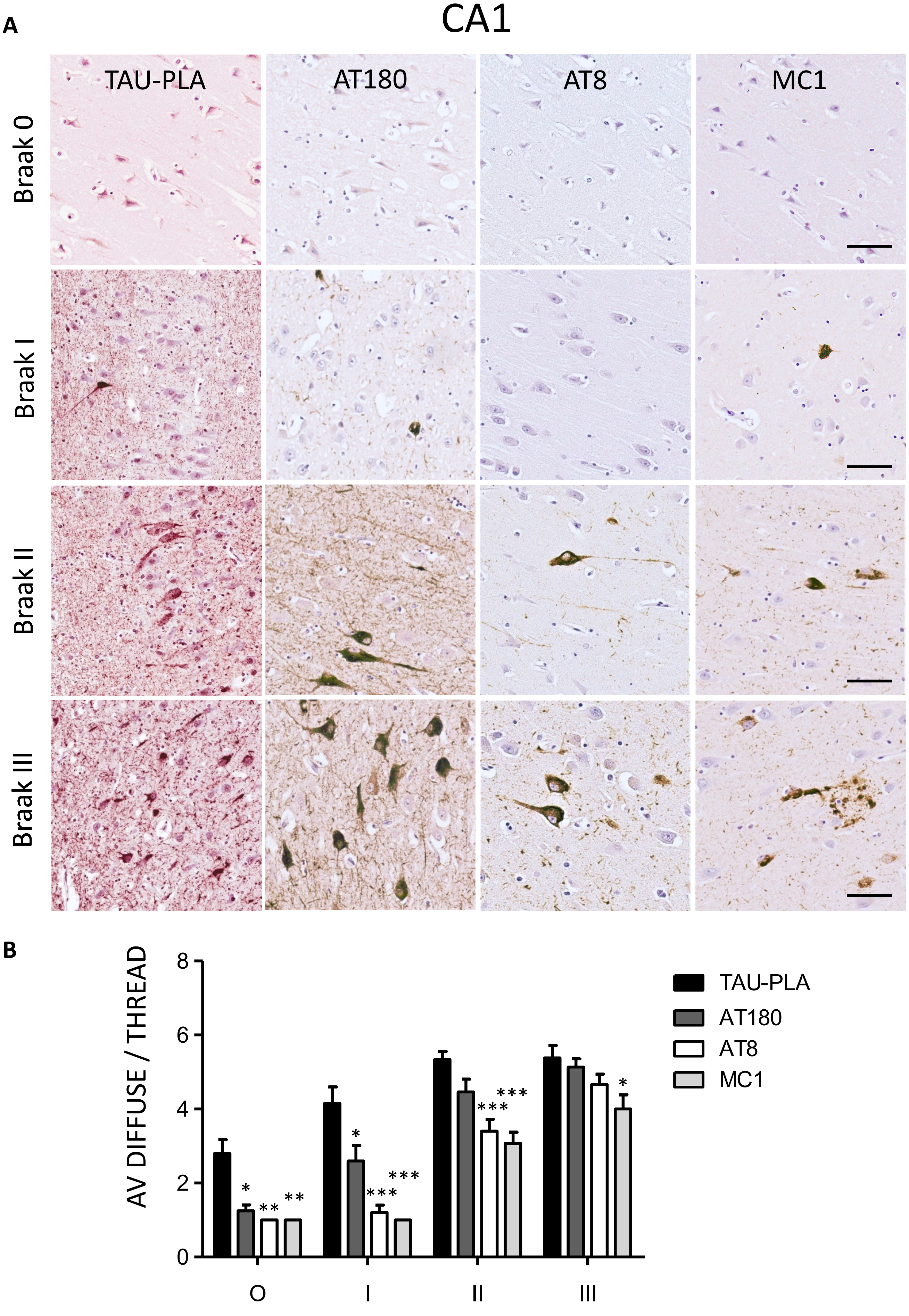

Figure S10. Early tau multimerization detection, prior to detection of tau hyperphosphorylation and misfolding across hippocampal regions and temporal isocortex– CA1.

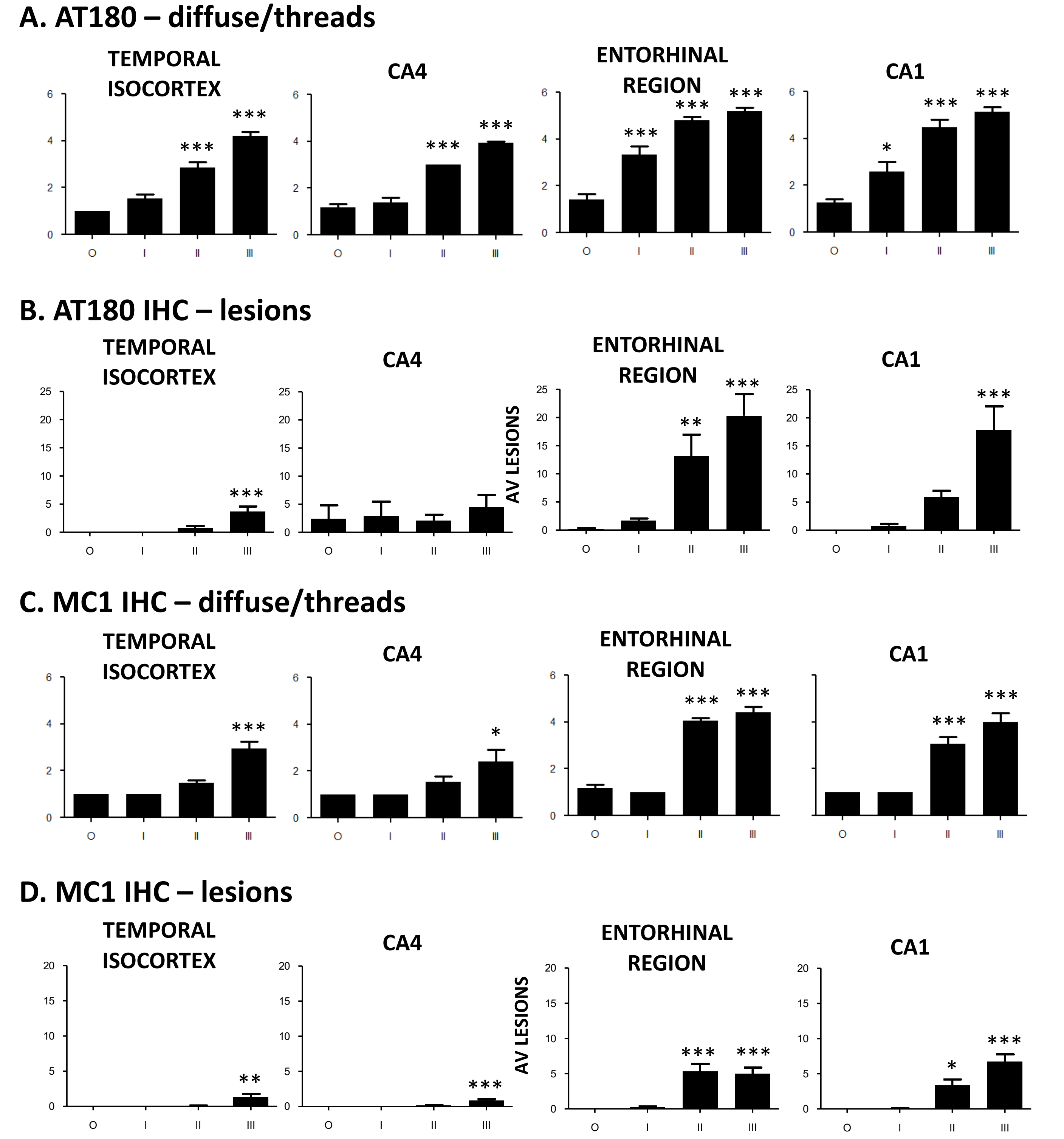

Figure S11. Quantification of diffuse pathology/threads and lesions labelled by AT180- and MC1- IHC in hippocampal regions and temporal isocortex.

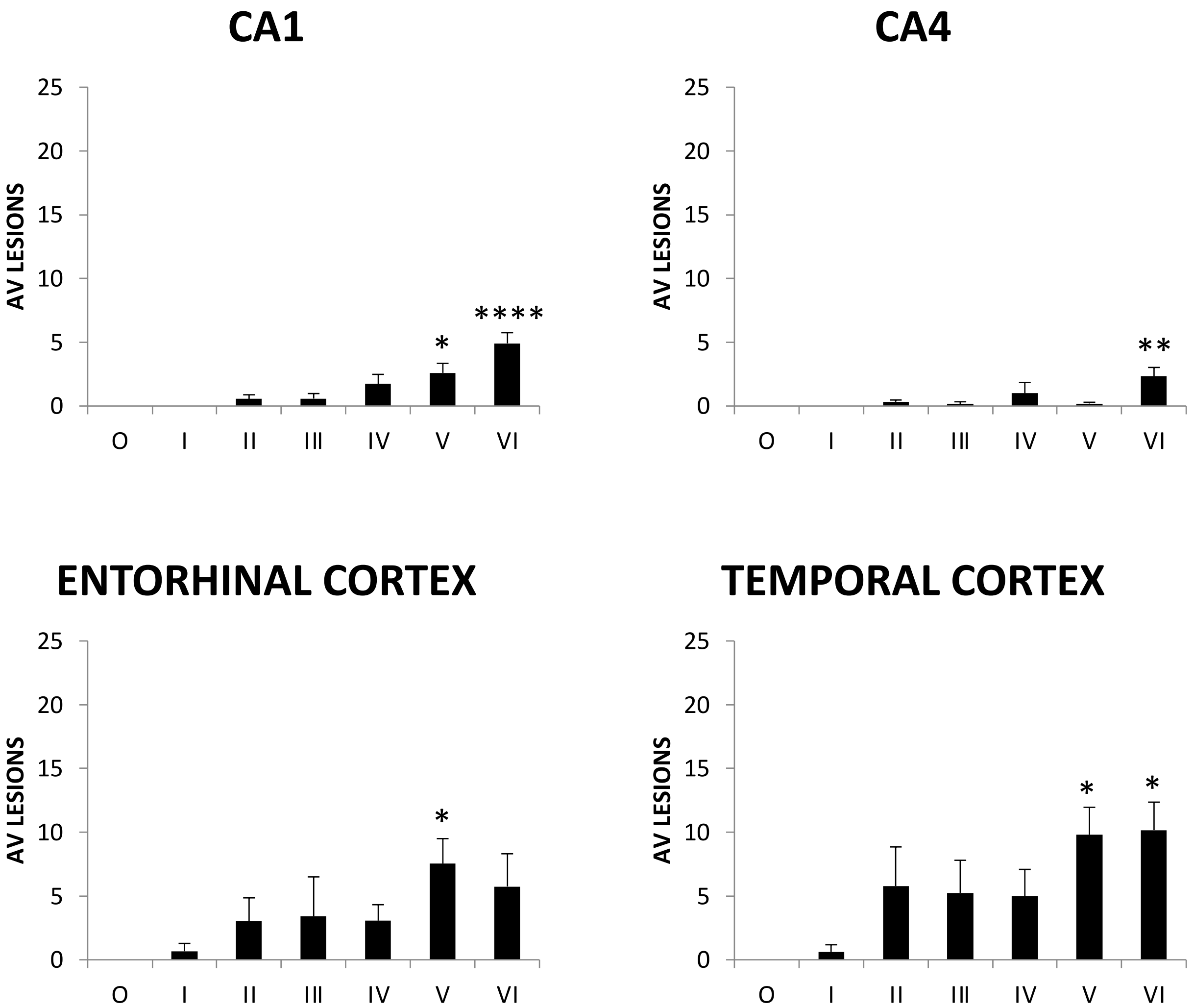

Figure S12. Quantification of lesions in samples labelled with 4G8 IHC.

Updated summary table (Oxford +Imperial cases)

	BRAAK 0	BRAAK I	BRAAK II	BRAAK III	BRAAK IV	BRAAK V	BRAAK VI
N=67	11	12	12	9	7	8	8
AV. AGE AT DEATH	54.6	68.7	82.6	89.1	87	85.7	76.5
% DEMENTED INDIVIDUALS	0%	0%	8.3%	11.1%	59.8%	100%	100%

Table S1. Summary of patient information

Updated table (Oxford +Imperial cases)

PMI	Dementia	Age at death	Gender	Amyloid burden (CERAD)	α-syn burden (Braak stage)	Neurofibrillary Braak stage
24	no	65	female	0, no neuritic plaques	0	0
48	no	42	female	0, no neuritic plaques	0	0
48	no	48	female	0, no neuritic plaques	0	0
72	no	51	male	0, no neuritic plaques	0	0
48	no	45	male	0, no neuritic plaques	0	0
48	no	41	female	0, no neuritic plaques	0	0
48	no	56	male	0, no neuritic plaques	0	0
19	no	74	male	0, no neuritic plaques	0	0
29	no	68	male	0, no neuritic plaques	0	0
20	no	52	female	0, no neuritic plaques	0	0
45	no	59	female	0, no neuritic plaques	0	0
48	no	56	male	0, no neuritic plaques	0	I
42	no	56	male	0, no neuritic plaques	0	I
65	no	92	female	0, no neuritic plaques	0	I
12	no	77	female	0, no neuritic plaques	0	I
100	no	88	female	0, no neuritic plaques	0	I
48	no	60	female	0, no neuritic plaques	0	I
48	no	51	male	0, no neuritic plaques	0	I
26	no	77	male	0, no neuritic plaques	0	I
19	no	91	female	1, sparse neuritic plaques	0	I
28	no	50	female	0, no neuritic plaques	0	I
15	no	61	female	0, no neuritic plaques	0	I
23	no	66	male	0, no neuritic plaques	0	I
54	no	92	female	0, no neuritic plaques	0	II
24	no	87	female	0, no neuritic plaques	0	II
48	no	57	female	0, no neuritic plaques	0	II
26	MCI	83	female	3, frequent neuritic plaques	1	II
24	no	89	female	1, sparse neuritic plaques	0	II
48	no	91	female	0, no neuritic plaques	0	II
96	no	79	male	0, no neuritic plaques	0	II
15	no	82	female	0, no neuritic plaques	0	II
26	no	83	male	2, moderate neuritic plaques	0	II
20	yes	82	female	2, moderate neuritic plaques	0	II
25	no	79	male	1, sparse neuritic plaques	0	II
15	no	87	female	2, moderate neuritic plaques	0	II
80	no	83	male	1, sparse neuritic plaques	0	III
86	MCI	90	female	0, no neuritic plaques	0	III
34	no	69	male	1, sparse neuritic plaques	0	III
72	no	92	male	1, sparse neuritic plaques	0	III
18	no	89	male	0, no neuritic plaques	0	III
24	no	92	female	3, frequent neuritic plaques	0	III
24	yes	98	female	3, frequent neuritic plaques	0	III
11	no	95	male	2, moderate neuritic plaques	0	III
40	MCI	94	female	3, frequent neuritic plaques	0	III
48	no	92	male	1, sparse neuritic plaques	0	IV
24	MCI	87	male	1, sparse neuritic plaques	0	IV
120	no	80	male	2, moderate neuritic plaques	0	IV
48	yes	89	male	3, frequent neuritic plaques	0	IV
12.75	yes	85	female	2, moderate neuritic plaques	0	IV
13	yes	89	female	3, frequent neuritic plaques	0	IV
28	no	95	female	2, moderate neuritic plaques	0	IV
31.25	yes	77	female	3, frequent neuritic plaques	0	V
46	yes	75	male	3, frequent neuritic plaques	Amygdala-only	V
66	yes	91	male	3, frequent neuritic plaques	0	V
34	yes	87	male	3, frequent neuritic plaques	0	V
24	yes	85	male	3, frequent neuritic plaques	0	V
45	yes	80	male	3, frequent neuritic plaques	0	V
34	yes	89	male	3, frequent neuritic plaques	0	V
21	yes	92	female	3, frequent neuritic plaques	0	V
7	yes	77	male	3, frequent neuritic plaques	0	VI
28	yes	64	female	3, frequent neuritic plaques	0	VI
13.67	yes	85	female	3, frequent neuritic plaques	0	VI
23	yes	61	female	3, frequent neuritic plaques	0	VI
69	yes	85	male	3, frequent neuritic plaques	0	VI
120	yes	83	male	3, frequent neuritic plaques	0	VI
7	yes	78	male	3, frequent neuritic plaques	0	VI
41	yes	79	male	2, moderate neuritic plaques	0	VI

Table S2. Additional information of brain samples.
